# The Effect of Orthology and Coregulation on Detecting Regulatory Motifs

**DOI:** 10.1371/journal.pone.0008938

**Published:** 2010-02-03

**Authors:** Valerie Storms, Marleen Claeys, Aminael Sanchez, Bart De Moor, Annemieke Verstuyf, Kathleen Marchal

**Affiliations:** 1 CMPG, Department of Microbial and Molecular Systems, Katholieke Universiteit Leuven, Leuven, Belgium; 2 Laboratory of Molecular Biology, Institute of Plant Biotechnology, Central University ‘Marta Abreu’ of Las Villas (UCLV), Santa Clara, Cuba; 3 Department of Electrical Engineering ESAT-SCD, Katholieke Universiteit Leuven, Leuven, Belgium; 4 Laboratory for Experimental Medicine and Endocrinology (LEGENDO), Katholieke Universiteit Leuven, Leuven, Belgium; The University of Chicago, United States of America

## Abstract

**Background:**

Computational *de novo* discovery of transcription factor binding sites is still a challenging problem. The growing number of sequenced genomes allows integrating orthology evidence with coregulation information when searching for motifs. Moreover, the more advanced motif detection algorithms explicitly model the phylogenetic relatedness between the orthologous input sequences and thus should be well adapted towards using orthologous information. In this study, we evaluated the conditions under which complementing coregulation with orthologous information improves motif detection for the class of probabilistic motif detection algorithms with an explicit evolutionary model.

**Methodology:**

We designed datasets (real and synthetic) covering different degrees of coregulation and orthologous information to test how well Phylogibbs and Phylogenetic sampler, as representatives of the motif detection algorithms with evolutionary model performed as compared to MEME, a more classical motif detection algorithm that treats orthologs independently.

**Results and Conclusions:**

Under certain conditions detecting motifs in the combined coregulation-orthology space is indeed more efficient than using each space separately, but this is not always the case. Moreover, the difference in success rate between the advanced algorithms and MEME is still marginal. The success rate of motif detection depends on the complex interplay between the added information and the specificities of the applied algorithms. Insights in this relation provide information useful to both developers and users. All benchmark datasets are available at http://homes.esat.kuleuven.be/~kmarchal/Supplementary_Storms_Valerie_PlosONE.

## Introduction

The identification of transcription factor binding sites (motifs) is crucial for the understanding of transcriptional networks. With the growing number of sequenced genomes [1]–[3], detecting motifs through ‘phylogenetic footprinting’ has become feasible. Several motif detection algorithms have therefore integrated the use of orthology in addition to the frequently used coregulation information [4]. Most of the original motif detection algorithms [5]–[12] could potentially incorporate orthologous sequences, but only by treating them independently and thus ignoring the underlying phylogeny that describes their relatedness. Because of this simplification, each orthologous sequence would contribute equally to the detected motif. This is counterintuitive as one would expect that a distantly related ortholog with a particular motif site contributes more information to the detection of that motif than a more closely related ortholog with the same site conserved. On the other hand, the loss of a motif site in a distantly related ortholog should be penalized less than when this loss event occurs in a more closely related ortholog [13]. A number of more recent probabilistic motif detection algorithms explicitly incorporate the relations between orthologous sequences by means of an evolutionary model, for example EMnEM [14], OrthoMEME [15], PhyME [16], the method by Li and Wong [17], Phylogibbs [18], Tree Gibbs Sampler [19] and Phylogenetic sampler [20].

So far no independent study has evaluated the extent of information contained within either the coregulation or the orthologous space and the conditions under which complementing both spaces improves motif detection. In this study we performed such analysis by applying two of the more advanced motif detection methods on both synthetic and real datasets with different properties. We choose for ‘Phylogibbs’ (PG) [18] and ‘Phylogenetic sampler’ (PS) [20] as both algorithms are specifically designed to integrate coregulation with orthology (therefore referred to as phylogenetic motif detection algorithms in this study), neither of them is limited in the number of species that can be included and previous studies [18] already described the superiority of PG in detecting motifs. As a comparison we included MEME [21] as a representative of algorithms that cannot explicitly incorporate phylogenetic relations (therefore referred to as a non-phylogenetic motif detection algorithm).

## Materials and Methods

### Motif Detection Algorithms and Parametersettings

Three motif detection algorithms were used: ‘MEME’ [21], ‘Phylogibbs’ [18], and ‘Phylogenetic sampler’ [20]. We used MEME-4.00 with default parameters, we set the distribution of motifs to “anr (any number of repetitions)” and the maximum number of EM iterations to 500. We searched for a palindromic motif (-pal) in case of TyrR and LexA for the real data (see Text S3). For PG we used Phylogibbs-1.0 and for PS we used Gibbs.x86_64. Before performing the tests on the synthetic and real datasets, we thoroughly tested the sensitivity of both algorithms towards parametersettings, not of primary importance for our main discussion, but that influence the results if not optimized. These tests and the optimized settings as applied in our analysis are summarized in Text S3. Most settings were not varied throughout the test runs except for the tracking threshold of PG that was set more stringent than its default value, unless indicated otherwise.

For PG prealignments were made with Dialign [22] (with the parameter T = 2 to avoid long unaligned regions obtained with higher values of T). For PS prealignments were obtained with ClustalW (version 1.83 [23]) as suggested by the developers. For the difficult to align datasets we also performed tests with PS on prealignments obtained with Dialign (results in Text S2). For those tests the results were similar or worse than those obtained with prealignments from ClustalW, indicating that the observed differences between PS and PG are caused by the intrinsically different way they cope with the prealignments rather than to small differences in the used prealignments. In general difficult to align sequences will be left unaligned with Dialign. This improves the alignment, but implies that those regions can no longer be used by PS (see also below). Therefore, for PS it is often more advantageous to use ClustalW instead of Dialign (which we therefore did in the remainder of the analysis).

### Synthetic Datasets

We created two synthetic motif weight matrices (WMs) as described previously [18], both of width 13 bp, one with a high information content (IC) and one with a lower IC. Motif sites sampled from these WMs were embedded at a randomly chosen position in a random background sequence of length 500 bp. Each ancestral sequence (∼a background sequence containing an embedded motif site) was then evolved along a phylogenetic tree under a defined evolutionary model to create phylogenetically related sequences. For the background sequence we used the Jukes and Cantor (JC) model [24], for the embedded motif sites an adapted Felsenstein (F81) model [25]. Details on the construction of the WMs and the evolutionary related sequences are in Text S1.

For the experimental setup we simulated datasets for the coregulation space, the orthologous space and the combined coregulation-orthology space. For the coregulation space, we simulated the intergenic sequences of ten genes in a reference species (the species exhibiting a proximity of 0.80 to the ancestral species was considered the reference species). In each of these 10 sequences a motif site, drawn from a common motif WM was embedded. For the combined space, we extended the coregulation space by simulating the orthologous intergenic sequences for each of the ten coregulated reference genes according to a phylogenetic tree that describes the relatedness of the orthologous sequences to the ancestral sequence. The topology of the phylogenetic tree was varied between a star topology (equal or unequal distances) and a tree topology with internal nodes. The orthologous space consisted of the intergenics of a single reference gene together with its simulated orthologs. For all trees used in our tests, the Newick format is given in Table S4.

### Real Datasets

The real datasets are derived from Gamma-proteobacterial and *Saccharomyces* intergenic sequences. Also here datasets were obtained with either a high IC or a low IC motif. For the coregulation space we selected target genes in *Escherichia coli* for the regulators LexA and TyrR and in *Saccharomyces cerevisiae* for the regulators URS1H and RAP1. To extend these datasets in the combined space, we searched for all target genes their corresponding orthologs in respectively other Gamma-proteobacterial or *Saccharomyces* species. The real datasets for the orthologous space only consist each time of one single target of the regulator in the reference species and its corresponding orthologs. In this case we selected as reference target, a gene that contained exactly one copy of the motif site in its upstream region in order not to confound coregulation with orthologous information (as the presence of multiple copies confers coregulation information). For the real data, we defined the upstream region as the intergenic region between the start codon of the gene and, depending on its orientation the start or stop of the previous coding gene. Details on the construction of the real datasets are in Text S1 and Table S2, the phylogenetic trees that relate the intergenic sequences of respectively the bacterial and yeast species are depicted in Figure S1. The Newick formats of the trees are given in Table S4.

### Performance and Quality Measures

#### Predicted motif sites

Motif sites predicted by MEME correspond to all sites obtained from the Expectation Maximization based solution. For PG, the ‘predicted motif sites’ are the motif sites from the tracked maximum *a posteriori* solution. For PS we defined the ‘predicted motif sites’ as the sites returned after running the ‘align-centroid’ option on the collection of centroid motif sites. More information on the output of PG and PS can be found in Table S1: ‘Solution/Posterior probabilities’. A ‘predicted motif model’ is the WM constructed from the predicted motif sites for a specific transcription factor.

#### Number of datasets/runs with an output (D1/R1)

For the synthetic data we had 100 input datasets per test. D1 gives the number of datasets for which the algorithm returned an output, irrespective of whether this output is correct or not. For the real data we only had one input dataset per test, so here we re-ran the algorithm ten times to get ten outputs for one input dataset. R1 represents the number of runs for which the algorithm returns an output. PG and PS internally evaluate their results and only report for each run or dataset the solutions that exceed a certain threshold. As a result for PG and PS, D1 and R1 sometimes are smaller than the number of runs. In contrast, MEME by default reports all retrieved results irrespective of their scores and therefore the number of datasets or runs with an output by definition equals the number of runs.

#### Recovery rate (RR)

The RR determines the percentage of the output (D1 for synthetic datasets) (R1 for real datasets), for which the predicted motif model corresponds to the ‘correct’ motif model. If a match is found between the predicted and the correct motif model, the recovery is one, otherwise zero. Motif models were compared with MotifComparison [26]. For the synthetic data the correct motif model was based on the embedded motif sites, for the real data on the annotated motif sites in the reference species (*E. coli* or *S. cerevisiae)*. Predicted models in the real data contain besides contributions from sites in the reference species also contributions from yet unannotated sites present in the orthologs. This sometimes causes discrepancy between the predicted and the correct motif model. For this reason predicted motif models that did not pass the MotifComparison threshold were retained if both the species-dependent positive predictive value and species-dependent sensitivity (for definitions see below) were above 50% or if one of the two measures was higher than 80%.

#### Positive predictive value (PPV) and sensitivity (Sens)

The PPV [PPV = TP/(TP+FP)] is a measure for the percentage of true positive (TP) sites amongst the predicted sites (TP+FP). A TP site corresponds in the synthetic datasets to the embedded sites and in the real datasets to the annotated sites. The false positives (FP) correspond to predicted sites, other than those embedded or annotated. The Sens [Sens = TP/(TP+FN)] is a measure for the percentage of true sites (TP+FN) that are found by the algorithm, with FN = false negatives corresponding to embedded or annotated sites not recovered by the algorithm. When a predicted site covers at least half the length of the embedded or annotated site, it is considered as a true positive site. For the less studied species other than *E. coli* or *S. cerevisiae* no judgement can be made on whether sites are true or false. Therefore, we defined the species-dependent PPV (spPPV) and species-dependent sensitivity (spSens) by only taking into account the sites predicted/annotated for the genes of the reference species. In the output tables of the Results section the PPV, spPPV, Sens and spSens are described per test and represent the mean of these values over all datasets/runs with a correct output (recovery equal to one) within a single test.

## Results

### Design of the Test Datasets

**Figure 1:**
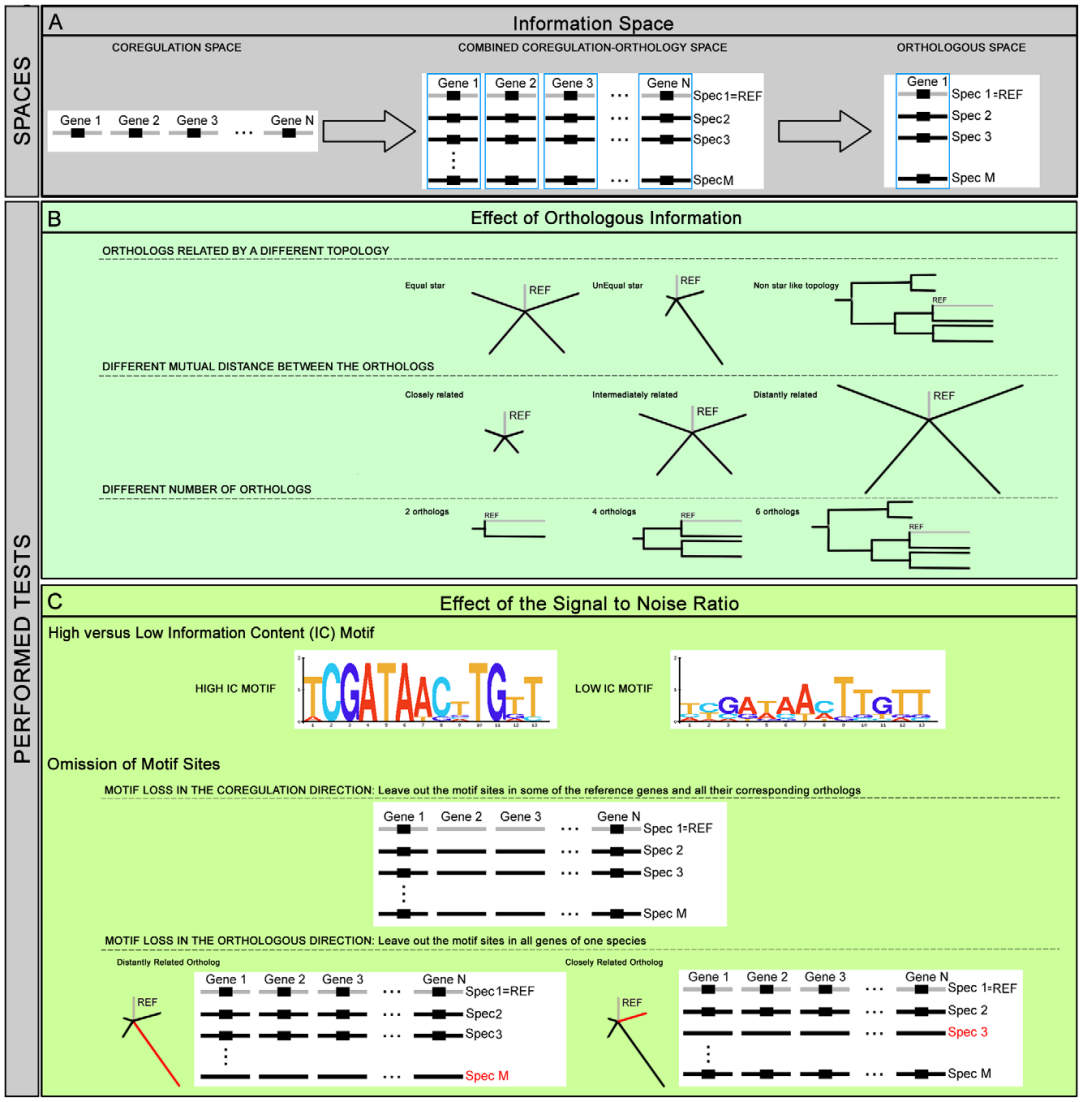
Overview of the test setup. Panel A presents the three different information spaces in which motif detection was assessed: the coregulation, the combined coregulation-orthology and the orthologous space. The coregulation space consists of a set of non-coding sequences from a reference species (Spec1 = REF) that each contain at least one motif site for a common TF (indicated by Gene 1 to Gene N). For the combined space, we extent the coregulation space with orthologous sequences selected from different species (indicated by Spec 2 to Spec M). One reference gene together with its orthologs is referred to as an *orthologous set* (indicated by a blue frame). The combined space thus consists of multiple orthologous sets while the orthologous space consists of a single orthologous set. We assessed the specific contribution of each space to the success rate of motif detection by performing the tests summarized in panels B and C. At first we tested the effect of adding different types of orthologous information as shown in Panel B. These tests involve changing the topology by which the orthologs are related (equal, unequal star and non star like topology), changing the mutual distance between the orthologs (represented by elongating the branches of the tree) and using datasets with a different number of orthologs. Secondly, the effect of altering the signal to noise ratio of the datasets on the accuracy of the results was tested 1) by changing the degree of degeneracy of the motifs and 2) by omitting motifs sites. We differentiate between leaving out motif sites in the coregulation direction versus their omission in the orthologous direction as is illustrated for a dataset in the combined space.

In this study we assessed the specific contribution of the coregulation, the orthologous and the combined space on motif detection (Figure 1 Panel A). Success rates observed in the coregulation space were treated as baseline levels. In the combined space we tested under which conditions adding orthologs improved the baseline success rate observed in the coregulation space. We tested the effect of changing the topology by which the orthologs are related, the phylogenetic distances and the number of the added orthologs (Figure 1 Panel B). Lastly, we evaluated the success rate of the algorithms when only orthologous information is available, also by using different conditions. In each space we performed tests on datasets with different signal to noise ratios (Figure 1 Panel C). We refer to ‘changing the signal to noise ratio’ as any manipulation that lowers/increases the degree to which the motif is statistically overrepresented in the dataset compared to the background e.g. by changing the degree of degeneracy of the motifs or by leaving out motif sites.

### Methodological Differences between the Used Algorithms

In all tests we observed that the motif detection results depend not only on the type of information that was added, but also on the interplay between the added information and the specificities of the applied algorithms. To view the results in the light of these algorithmic characteristics, we here outline the most important differences between the applied algorithms. A more detailed comparison can be found in Table S1.

At first, PG and PS are both based on Markov Chain Monte Carlo (MCMC) sampling [27] to efficiently explore the solution space. To converge to a global solution in a single run of the algorithm, PG relies on a specifically designed optimization strategy, while PS estimates the global solution by combining local solutions obtained from different runs into a final ensemble centroid solution [28]. MEME on the other hand uses an Expectation Maximization strategy that often leads to a local rather than a global optimum.

In contrast to MEME, PG and PS explicitly model the phylogenetic relatedness between the orthologous sequences. They do so by scoring motif sites that are located in evolutionary conserved regions with a tree-based evolutionary model. These evolutionary conserved regions are delineated in advance by means of a prealignment. PS only considers the regions that are aligned (conserved) over all input sequences (∼blocks). It does not search for motifs in any of the regions that contain gaps, even if those regions are aligned over a subset of the sequences. PG in contrast, does consider the complete sequence alignment when searching for motifs. It scores motif sites in the aligned subparts (∼multi-species window) phylogenetically, while treating the motif sites in the unaligned parts (∼single-species window) independently. This different way of treating the prealignment implies the need of different alignment strategies to delineate evolutionary conserved regions prior to the actual motif detection. PS relies on a global alignment as obtained by ClustalW [23] to identify the conserved blocks, while the more refined procedure of PG requires an alignment strategy, such as Dialign [22] that explicitly annotates aligned and unaligned regions.

A third difference relates to the scoring of the evolutionary conserved motif sites by the tree-based evolutionary model; here PS handles a non star like topology directly, while PG can not.

Finally, PS accounts for the phylogenetic relatedness also during the construction of the motif WM by means of a weighting scheme, while PG does not. This weighting scheme assigns a higher weight to motif sites conserved in distant orthologs in their contribution to the motif WM than to sites conserved in close orthologs.

### Motif Detection in the Coregulation Space

**Figure 2:**
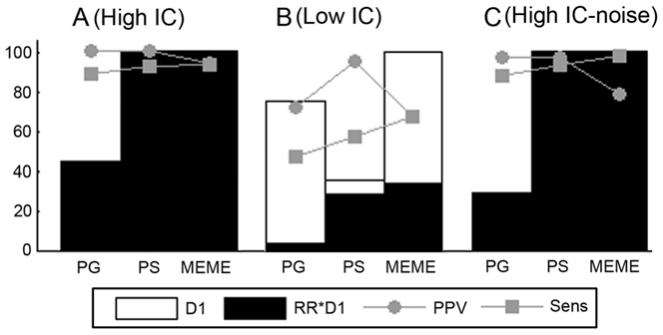
Results for motif detection in the coregulation space. Each dataset consists of ten coregulated genes from the reference species (proximity 0.80). Panel A displays the results for a synthetic dataset in which all sequences contain a site sampled from a high IC motif (A). Panel B shows the results for a dataset in which all sequences contain a site sampled from a low IC motif (B) and panel C shows the results of a dataset where the motif site is missing in two out of ten sequences. The remainder of the sequences contains a motif site sampled from the high IC motif. Results were assessed by the performance measures D1: the number of datasets with an output out of 100 datasets, D1*RR: the number of datasets with a correct output and the quality measures PPV (the percentage of true sites among the predicted motif sites, averaged over all correct outputs) and Sens (the percentage of the true sites recovered by the algorithm, averaged over all correct outputs).

Datasets consist of sets of coregulated genes from the reference species. We tested the ability of the algorithms to recover motifs in datasets with different signal to noise ratios. The most trivial task consists of detecting a high IC motif in a dataset where each sequence contains a motif instance (Figure 2(A)). We also assessed whether the motif detection tools could recover motifs in datasets with lower signal to noise levels e.g. by searching for a low IC motif (Figure 2(B)) or by searching for a high IC motif in a dataset where not all sequences contain a motif instance (Figure 2(C)). We applied those tests on both synthetic and real datasets. Figure 2 summarizes the results for the synthetic datasets (from Table S5 (A) and Table S6) as these reflect the most important tendencies. Details on the results for the real datasets can be found in Table S5 (D).

Results were evaluated by ‘performance measures’ and ‘quality measures’. The ‘performance measures’ describe whether the motif detection tool is able to retrieve the motif model of the embedded motif in a particular test. They correspond to *the number of datasets with an output* (D1) and the *recovery rate* (RR) that indicates the percentage of outputs in which a correct motif was predicted. The ‘quality measures’ defined as the *positive predictive value* (PPV) and the *sensitivity* (Sens) describe whether and how many of the true embedded motif sites contribute to the predicted motif model. In the figures the number of datasets with an output (D1) is indicated by a clear box. The number of those datasets that has a correct outcome (D1*RR) is indicated by the black area in the clear box. A larger fraction of the black area in the box (RR) indicates that a larger fraction of the output is correct. The best results are thus obtained if most of the outputs contain a true motif model (largely filled boxes) of a high quality (the latter is indicated by the PPV and Sens approaching 100).

In Figure 2 we see that the results were consistent for the three tested algorithms. It shows that coregulation information is sufficient to detect the correct motif provided that the motif has a high IC. For a low IC motif, both the RR and the motif quality (assessed by PPV and Sens) drop. More specifically we had to lower the tracking threshold T of PG to 0.05 in order to still retrieve this low IC motif. Lowering the tracking threshold results for PG in general, in a higher number of datasets with an outcome (D1), but at the cost of a decreased RR and PPV. Of the three algorithms tested, PS performed best for these low IC motifs with a RR equal to of 80.6%, compared to a RR of 34% for MEME and 5.3% for PG. As shown in Figure 2(C), all algorithms are quite robust against the presence of sequences without motif site provided the motif itself is sufficiently pronounced. Based on these results, we expect that including orthologous information will be beneficial if it increases the signal to noise ratio in the dataset e.g. when searching for a low IC motif.

### Motif Detection in the Combined Coregulation-Orthology Space

In this section we assessed to what extent adding orthologous information to the coregulation space improves motif detection. For the algorithms that rely on a phylogenetic model we expect that their results will depend on the accuracy with which the used phylogenetic tree approximates the true phylogenetic distances between the used intergenic sequences. For real data approximating an optimal tree is not obvious as the intergenic sequences can not accurately be aligned. The best results were obtained with a tree that is based on a ‘neutral’ evolution rate. Using a protein tree seriously deteriorated the results obtained by the phylogenetic motif detection algorithms as the true evolution rate of the intergenic sequences is underestimated (for more details see Table S3). In all tests, we used for the phylogenetic algorithms the tree based on a neutral evolution rate. If the input sequences were left unaligned, PG and PS will just like MEME treat the sequences independently.

#### Effect of the phylogenetic distances between the orthologs

Datasets consist of coregulated genes in the reference species (coregulation space) complemented with their respective orthologs (orthologous space). A reference gene together with its orthologs constitutes an orthologous set. For the first set of tests, the relatedness between the sequences in an orthologous set was modeled by a *‘star topology with equal distances’*. Each orthologous set consists of the reference sequence (proximity of 0.80) and four *equally* distant orthologs. The tests consist of changing the distance (∼“proximity”) for these four orthologs that were added to each coregulated reference gene.

**Figure 3:**
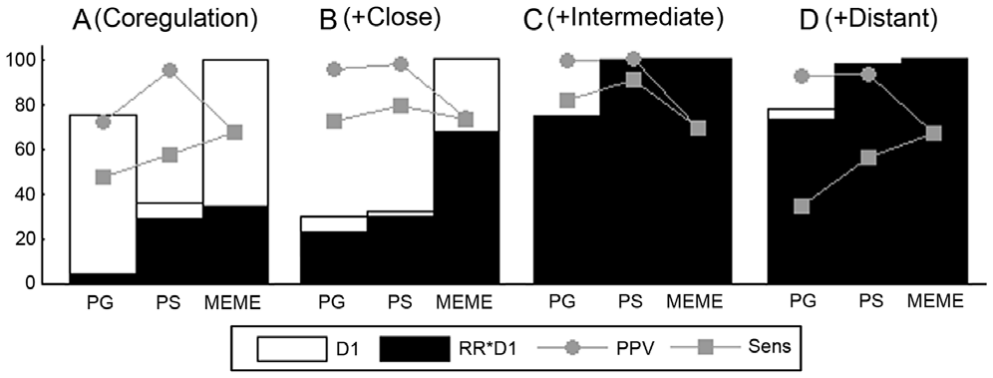
Effect of adding orthologs with distinct phylogenetic distances on motif detection in the combined space. Results are displayed on the retrieval of a low IC motif in a synthetic dataset. Panel (A) shows the results for the coregulation space that consists of ten coregulated reference genes. The remaining panels represent the results for the combined space that consists of the ten coregulated reference genes together with their orthologs, also referred to as ten orthologous sets. Each orthologous set consists of five prealigned sequences related through an equal star topology: the reference sequence with proximity 0.80 and four equally distant sequences with proximities of respectively 0.90 (B), 0.50 (C) and 0.20 (D). For the measures D1, D1*RR, PPV and Sens see Figure 2.

All results for a high and low IC motif resumed in Table S5 (A) reflect the same tendency, summarized for one representative example in Figure 3. Figure 3 shows how the detection of a low IC motif was affected by adding to a set of coregulated reference genes (Figure 3(A)), either closely related orthologs (proximity of 0.90, Figure 3(B)), intermediately (proximity of 0.50, Figure 3(C)) or distantly related orthologs (proximity of 0.20, Figure 3(D)). Adding orthologous information improved the RR for all algorithms (fraction of the black area). The best results were obtained for a proximity of 0.50 (Figure 3(C)) and under these optimal conditions, algorithms that use an evolutionary model clearly outperform the non-phylogenetic motif detection algorithm in finding high quality motifs (both Sens and PPV). All algorithms are sensitive towards deviations from the optimal phylogenetic distance between the added orthologs. Too closely related orthologs (Figure 3(B)) imply many local optima and this resulted for all algorithms, compared to the more optimal situation, mainly in a decrease of the RR. This drop in RR was most obvious for MEME as it does not use an evolutionary model. PS performed best (highest RR) for these datasets where the motif is less pronounced. Adding orthologs that were all very distantly related (Figure 3(D)) was mainly deleterious for the phylogenetic algorithms as they depend on the quality of the prealignments: misalignment of motif sites or gaps introduced within the sequence of the motif sites make it harder or even impossible to retrieve these motif sites, which resulted in a lower motif quality (especially a lower Sens) for PG and PS compared to MEME. In some cases leaving the distant orthologs unaligned can compensate for the loss in sensitivity (Table S5 (A)).

In a second set of tests, we examined if adding one distantly related ortholog to a set of closely related orthologs reduces the number of local optima and hence improves the motif detection results. To this end we used for each coregulated reference gene an orthologous set for which the relatedness was modeled by a *‘star topology with unequal distances’*. Each orthologous set consists of four closely related orthologs with proximities of respectively 0.80 (the ortholog of the reference species), 0.90, 0.85 and 0.75 and one distantly related ortholog with a proximity of 0.20. All results represented in Table S5 (B) confirmed our expectation: compared to using orthologous sets containing only the four closely related orthologs, adding one distantly related ortholog to the orthologous set of each coregulated reference gene improved the RR of all algorithms. For the phylogenetic algorithms the number of datasets with an output (D1) increased, especially for the low IC motif. The increase in RR was sometimes at the expense of a small sensitivity (Sens) loss for the predicted motif, which was mainly caused by the algorithms not being able to detect the motif sites in the distant orthologs. This was confirmed by specifically calculating the sensitivity in the distant ortholog (species-dependent sensitivity, spSens) which was indeed lower than the overall sensitivity (results in Table S5 (C)). In this example where the synthetic datasets were particularly easy to prealign (equal sequence lengths), including the distant ortholog in the prealignment of the orthologous sets improved the results of both phylogenetic algorithms.

#### Effect of the number of added orthologs

**Figure 4:**
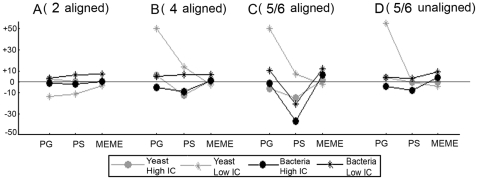
Effect of the number of added orthologs on motif detection in the combined space. Results on the retrieval of both a high and a low IC motif are displayed for the real datasets: 1) results from the Gamma-proteobacterial datasets are indicated as black curves and 2) those of the *Saccharomyces* dataset are indicated as gray curves. Results for the high IC motif are indicated by circles and correspond to those obtained for LexA (bacterial dataset) or URS1H (yeast dataset), results for the low IC motif are indicated by stars and correspond to those obtained for TyrR (bacterial dataset) or RAP1 (yeast dataset). The panels represent the results of a dataset containing for each coregulated reference gene two (A), four (B) and six (for the bacterial datasets) or five (for the yeast datasets) prealigned orthologs (the reference gene included) (C). Panel (D) represents the results of a dataset containing for each coregulated reference gene six or five unaligned orthologs (the reference gene included). Results were assessed by the F-value defined as the harmonic mean of the spPPV (the percentage of true sites amongst the predicted motif sites for the reference species, averaged over all correct outputs) and the spSens (the percentage of the true sites found by the algorithm for the reference species, averaged over all correct outputs). The reference species are respectively *E. coli* (bacterial data) or *S. cerevisiae* (yeast data). The Y-axis represents the difference between the F-value obtained from searching motifs in the combined coregulation-orthology space and the F-value obtained from searching in the coregulation space only.

For each dataset, we started off with a real set of coregulated genes in the reference species (the target genes of respectively LexA, TyrR in *E. coli* and URS1H, RAP1 in *S. cerevisiae*) and tested the effect of gradually adding more distant orthologs to these reference genes. All results are shown in Table S5 (D). As for most tests the performance parameters (R1 and RR) reached their maximum level, the most striking results for both the bacterial and yeast datasets relate to changes in motif quality. To visualize this tendency in motif quality observed for both the bacterial and yeast datasets we used a combined ‘quality’ metric, the F-value, defined as the harmonic mean of spPPV (species-dependent PPV) and spSens (species-dependent sensitivity). Figure 4 displays the difference between the F-value obtained from searching in the combined coregulation-orthology space and the F-value obtained from searching in the coregulation space only. The results are shown for datasets in which for each coregulated gene the orthologous sets contain respectively two (Figure 4(A)), four (Figure 4(B)), five (yeast)/six (bacteria) prealigned orthologs (Figure 4(C)), and five/six unaligned orthologs (Figure 4(D)). A positive value of the F-value difference thus indicates a positive effect on the motif quality of adding orthologs to the coregulation space, while a negative value indicates the negative effect.

In general the results confirm what we already observed for the synthetic data (see previous section: ‘Effect of the phylogenetic distances between the orthologs’): at first, adding orthologous information has more impact on the results when searching for a low IC motif than when searching for a high IC motif. Adding orthologs barely improved the motif quality when searching for a high IC motif (LexA and URS1H) (Figure 4).

Secondly, the quality of the motifs retrieved by the phylogenetic tools is more sensitive towards the type of orthologs that was added than MEME because their results depend on the correctness of the prealignments. Figure 4(C) shows that for PS, the F-value difference dropped drastically when adding the more distantly related orthologs that can no longer be accurately aligned with the closely related ones. The effect was more pronounced for the bacterial datasets that were the most difficult to prealign. As a result leaving all orthologs unaligned in those cases of misalignment (Figure 4(D)) improved the quality of the motifs retrieved by PS. All four panels in Figure 4 show that for MEME, the effect of adding orthologs on the quality of the retrieved motif is rather small.

Additional tests on synthetic data (see Table S7) ensured us that the differences in performance between the motif detection algorithms we observed when adding orthologs could indeed be attributed to the gradually increased phylogenetic relatedness between the added orthologs, rather than to the intrinsically different way PG and PS handle non star like topologies in their phylogenetic model.

#### Simulation of motif loss in the orthologous and coregulation direction

**Figure 5:**
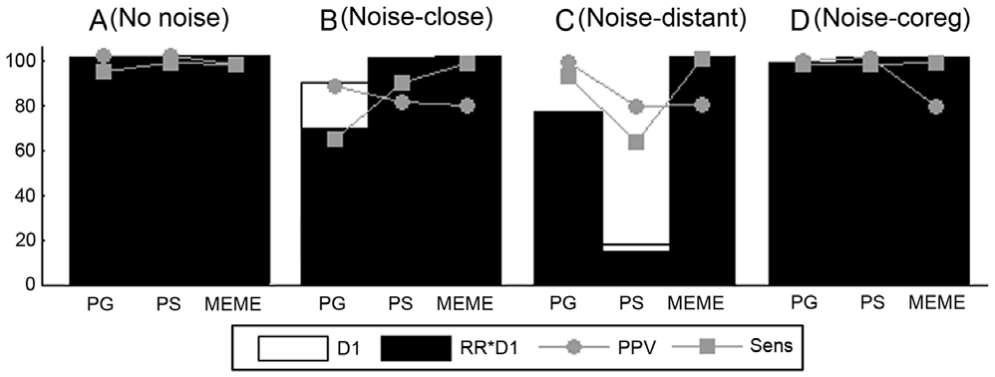
Effect of motif loss on motif detection in the combined space. The results are displayed for a synthetic dataset containing sites sampled from a high IC motif. Each dataset consists of ten coregulated reference genes complemented with their orthologs, also referred to as ten orthologous sets. Each orthologous set consists of five prealigned sequences related through an unequal star topology: four closely related orthologs with proximities of respectively 0.80 (reference ortholog), 0.90, 0.85 and 0.75 and one distantly related ortholog with a proximity of 0.20. Panel (A) represents the results when a motif site is present in all sequences of the orthologous sets. Panels (B) and (C) display the results when motif loss occurs in all sequences derived from respectively a closely (q = 0.75) or a distantly (q = 0.20) related species. Panel (D) shows the results when motif loss occurs in two out of ten coregulated reference genes and in all their corresponding orthologs. For the measures D1, RR*D1, PPV and Sens see Figure 2.

Previous tests showed that adding orthologs was beneficial, provided that they contain the motif site. However, adding orthologous sequences from species in which the mode of regulation is not conserved will increase the noise in the input datasets [29], [30]. Here we simulated this situation by adding orthologs to a set of coregulated reference genes, but assuming that all added sequences derived from one species did not contain the motif site. The relatedness between the sequences in the orthologous sets was modeled by a star topology with unequal distances. Figure 5 summarizes these results for a high IC motif (as given in Table S6). Figure 5(A) shows the reference level of performance when a motif site is present in all sequences of the orthologous sets. In the remainder of the panels the results are shown of replacing in each orthologous set the motif site by a random site in the sequence derived from either a closely related species (proximity 0.75, Figure 5(B)) or a distantly related species (proximity 0.20, Figure 5(C)). As shown in Figure 5(B and C), all three algorithms were affected by adding orthologs without motif site. For PG the absence of the motif sites in closely related orthologs (Figure 5(B)) had a more pronounced negative influence (drop in RR, PPV and mainly Sens) than when the motif site was absent in the distantly related orthologs (Figure 5(C)). For PS the situation is reversed: the presence of distant orthologs without motif site resulted in a drastic drop in D1 and in the Sens compared to the reference situation (where the motif site was present in all orthologs) (Figure 5(A)) or to the situation where the motif site was absent in the closely related orthologs (Figure 5(B)). The difference in response between PG and PS towards the absence of motif sites is related to the intrinsically different way they treat the prealignments (see also Table S6 for more information). When the motif site is missing in the distant orthologs, regions that normally would contain the motif site will be left unaligned by Dialign or will result in a gapped alignment by ClustalW. In neither case PS will search for motifs in these regions of the prealignment while PG will correctly treat these regions independently and search for motifs in the remaining part of the prealignment. Missing motif sites in the close orthologs on the other hand are better handled by PS as it relies on a global alignment strategy. As closely related orthologs align any way well over the total length of their sequence, a global alignment is not too much disturbed by a missing motif site in one of these close orthologs. For a local alignment this often interferes with the correct identification of the orthologous regions.

In addition, for PS also the weighting scheme used during the update step of the motif WM affects its specific behavior towards missing motif sites in distantly related sequences. Distantly related orthologs get a higher weight than closely related ones, so a false positive site in a distant ortholog has a more negative impact on the WM update than a false positive site in a close ortholog.

For MEME mainly the motif quality (more in particular the PPV) was decreased by omitting motif sites, but in contrast to what was observed for the phylogenetic algorithms this effect was largely independent of the type of ortholog from which the sites were omitted (Figure 5(B, C)). By setting the number of asked motif sites equal to the number of input sequences, the number of sites we searched is overestimated when leaving out motif sites. This effect of overestimating the number of motif sites affects the quality of the motif retrieved by MEME that does not internally filter out low quality motif sites.

As for the coregulation space, we also tested for the combined space the effect of missing motif sites in *the coregulation direction.* This situation was mimicked by assuming that two of the reference genes were not truly coregulated with the other genes. The motif site is thus absent in these two genes and in their respective orthologs. Figure 5(D) shows that this had almost no effect on the results, except for a PPV drop in case of MEME with the same reason as above.

Figure 5(D) also shows that omitting motif sites in the coregulation direction has less drastic effects on the results (most obvious for PG) when also the orthologs are provided than in the absence of the orthologs (Figure 2(C)), even though some of the orthologs might not contain the motif site.

### Motif Detection in the Orthologous Space

Lastly we assessed the performance of the algorithms in the presence of only orthologous information. We used a test setup similar as in the combined coregulation-orthology space, but instead of using a set of coregulated reference genes complemented with their orthologs, we used only one reference gene together with its orthologs (∼one orthologous set). The tests consist of changing for this orthologous set the number of orthologous sequences and their phylogenetic relatedness (equal or unequal star topology). We also assessed in real datasets the effect of gradually adding more orthologs with increasing phylogenetic distance to the orthologous set.

**Figure 6:**
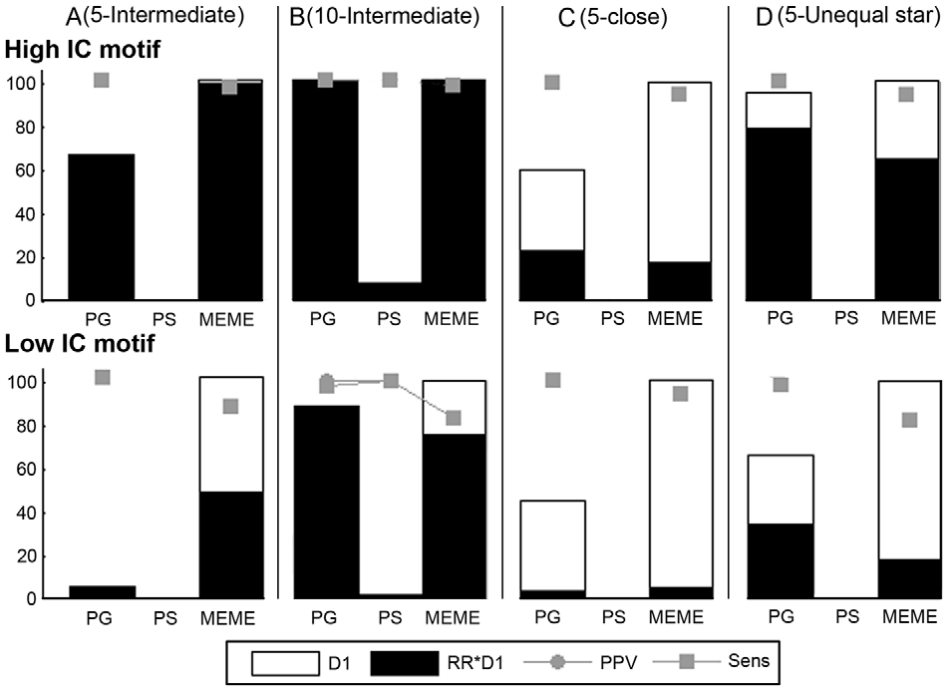
Results for motif detection in the orthologous space. Results are displayed for a synthetic dataset with motif sites sampled from a high IC (on top) and a low IC motif (below). Each dataset consists of only one reference gene and its orthologs, referred to as one orthologous set. Panel (A) and (B) represent the results when the orthologous set contains respectively five and ten prealigned orthologs related through an equal star topology with a proximity of 0.50. Panel (C) represents the results when the orthologous set contains five prealigned orthologs related through an equal star topology with a proximity of 0.90 and panel (D) represents the results when the orthologous set contains five prealigned orthologs related through an unequal star topology. Note that for most tests the PPV equaled the Sens resulting in overlapping dots. For the measures D1, RR*D1, PPV and Sens see Figure 2.

All results for the synthetic data are shown in Table S8 (A). Figure 6 shows representative results for the tested algorithms in detecting respectively a single embedded high (at the top) and low (at the bottom) IC motif. Figure 6(A) and (B) show the results for the orthologous set containing respectively five and ten orthologs related through an equal star topology with proximity 0.50. Figure 6(C) shows the results for the orthologous set containing five orthologs related through an equal star topology with proximity 0.90 and Figure 6(D) shows the results for the orthologous set containing five orthologs related through the earlier described unequal star topology (see previous section: ‘Motif detection in the combined space’). All algorithms performed best on datasets with 10 prealigned orthologs related to each other with a proximity of 0.50 (Figure 6(B)). For this setting, PG and PS outperformed MEME (higher RR and motif quality), especially for the low IC motif. However, for PS the number of datasets with an output was extremely low (D1<10). By keeping track of the motif positions sampled during the early iteration stage of PS, we noticed that the sampler explored the solution space less for the prealigned input than when leaving the sequences unaligned. By getting stuck in non-overlapping local optima for each re-initialization, no centroid output could be obtained (low D1). The performance of all algorithms dropped when the number of prealigned orthologs was lowered to 5 (Figure 6(A, C and D)) in which case PS even did not longer retrieve an output. Using too closely related orthologs (Figure 6(C)) resulted in a severe further decrease of the RR for both MEME and PG (despite lowering the tracking threshold). As was also the case in the combined space, we can increase the information level of the datasets by adding one distant ortholog through the use of a ‘star topology with unequal distances’ (Figure 6(D)): this improved the performance (D1 and RR) of both PG and MEME considerably compared to the situation with closely related orthologs of equal phylogenetic distance.

For the real datasets, we used two reference targets genes of LexA, two of TyrR, two of URS1H and two of RAP1 each containing exactly one motif site for their respective regulators and we added to each of these individual genes their orthologs resulting in 8 datasets in total. As was done in the combined space, these orthologs were added gradually with increasing phylogenetic distances. The results on the real datasets in the orthologous space (Table S8 (B)) were rather poor and similar to what was observed for the synthetic data: when the dataset contains too few closely related orthologs (less than six for bacterial genes and four for the yeast genes) the algorithms failed in detecting the motif (data not shown). Increasing the information level of the datasets by adding extra orthologs resulted in PG and MEME becoming able to retrieve the motif for at least some of the datasets. For PG the best results were obtained by including the phylogenetic relatedness by means of a prealignment. PS totally failed on the real data in the orthologous space, even for the maximum number of orthologs (irrespective of whether they were aligned or left unaligned).

## Discussion

In this work, we tested the impact of using coregulation and/or orthologous information on the efficiency of regulatory motif discovery by two representative motif detection algorithms with an evolutionary model. We designed appropriate benchmark datasets and made an exhaustive evaluation of both algorithms together with MEME, a well-known reference algorithm. Parameter tuning required a detailed analysis of how parameters influence test results. This analysis (see also Text S3) together with guidelines describing how the selection of the best tool depends on the composition of the dataset is summarized in Table 1.

**Table 1:** Summary of user-guidelines.

PROBLEM	CONSTRUCTING DATASET	PREFERRED TOOLS	REMARKS
1. COREGULATION SPACE			
Maximizing the signal to noise ratio in the dataset (i.e. the enrichment of motif sites in the dataset) improves the success rate.	Only select sequences that are likely to contain the motif. Keep the input sequences as short as possible.Adding orthologs (see 2: combined space) improves the success rate at a low signal to noise ratio.	PS: the ensemble centroid solution guarantees a high success rate for datasets with low signal to noise ratios. MEME: easy to use with performances comparable to those of PG and PS.	Both PG and PS provide a statistical procedure to filter out non-significant motif sites = > Overestimating the ‘expected number of motif sites’ affects the performance less than underestimating them. For MEME misestimating the expected number of motif sites affects the motif quality.
2. COMBINED SPACE			
It is crucial to use a **phylogenetic tree** that reflects the true evolutionary distances between the intergenic sequences.	Use a tree based on a neutral evolution rate or a protein tree with corrected distances to prevent underestimating the evolution rate.	Both PG and PS are sensitive to overestimating the evolutionary proximity of the orthologous intergenic regions.	The type of topology (star, tree like structure) does not affect the performance of the phylogenetic tools.
The **characteristics of the added orthologs**: mainly the **evolutionary distance** between them influences the results by affecting the trade-off between align-ability of the orthologs and the information level of the dataset.**Close orthologs**: the dataset contains little information**Intermediate orthologs:** this is the optimal situation.**Distant orthologs**: the dataset contains more information, but the alignment might get deteriorated.	**Close** ∼ q = 0.90, the orthologs align for almost 100%. In this case add at least one distant ortholog to increase the information level of the dataset.**Intermediate** ∼ q = 0.50, the sequences can be aligned and contain sufficient information (clear phylogenetic shadowing of the motif).**Distant** ∼ q = 0.20, the prealignment looks bad. For the phylogenetic tools it is better to leave the difficult to align sequences unaligned.	**Close**: the phylogenetic tools outperform MEME because of the multiple local optima in the data.**Intermediate**: for this optimal evolutionary distance the phylogenetic tools outperform MEME.**Distant**: an unreliable prealignment deteriorates the results of the phylogenetic tools. MEME performs better under those conditions. In general PG better handles these difficult to align datasets than PS.	The **number of orthologs** to be added is of less importance for the success rate. Good results can already be obtained with 4 orthologs, provided that they have a good evolutionary distance.**PG is easier to use than PS:** 1) when the dataset contains a different number of orthologs per gene, PG adapts the input phylogenetic tree automatically while for PS it needs manual interference. 2) PS has a long running time compared to PG and MEME.
**Motif Loss** in a **closely** related ortholog or in a **distantly** related ortholog increases the noise in the dataset.	Avoid sequences for which one expects that the mode of regulation has changed (mostly the distantly related sequences).	PG/PS performs better if the motif is omitted in the distant/close ortholog. MEME: not dependent on the type of ortholog.	
3. ORTHOLOGOUS SPACE			
The same issues as in 2 are valid regarding the phylogenetic tree and the characteristics of the orthologs.	The more orthologs are added, the better the results.	PG performs best when the orthologs are prealigned and slightly outperforms MEME. PS underperforms in the orthologous space.	Observing a PG output that only contains unaligned motif sites indicates that the input tree underestimates the true evolution rate. In that case, lower the proximities.

From our results it appeared that coregulation data allow all three motif detection algorithms to retrieve the motif if the signal to noise ratio in the data is high. In real life situations it is more common to encounter datasets with a low signal to noise ratio, as biologists often define coregulated gene sets based on results derived from noisy high throughput experiments. Moreover the length of the intergenic sequences can be long compared to the length of the motif sites [31] and often the motifs themselves are heavily degenerated. Under such conditions, adding orthologous information to the coregulation space can improve the results. There seems to exist an optimal phylogenetic distance between the added orthologs, for which all algorithms retrieved the best results. This optimal distance corresponds to orthologs that are still alignable, but show a sufficient level of divergence so that non functional background sequences are no longer conserved and the signal of the conserved motif site stands out in the background sequence. For applications of phylogenetic footprinting, where motifs are searched for in the orthologous space, there is still room for improvement. All three algorithms performed poor, partially because they were originally developed and tuned towards searching for motifs in the coregulation or the combined coregulation-orthology space.

In all tests we observed some reoccurring effects that can be explained by the algorithmic specificities of the applied motif detection algorithms.

At first we consistently observed that PS outperforms PG and MEME when the signal to noise ratio drops in the dataset. This is because PS uses an ensemble of solutions to define the statistically most overrepresented motif in the dataset whereas both PG and MEME report a single optimal solution. Especially in the presence of multiple local optima, such ensemble strategies have proven to be more successful in estimating the true optimum than searching for a single optimal solution [32]. However, this advantage of using an ensemble solution comes at the expense of much longer running times (e.g. a dataset with 10 orthologous sets each containing five orthologs had a running time around 8 hours, for PS, compared to several minutes for PG and MEME).

Secondly, we would expect that modeling the relation between orthologous sequences when searching for motifs in the combined or the orthologous space would improve results over those obtained with MEME, or with PG and PS when leaving the sequences unaligned. Using an evolutionary model in combination with a tree that correctly represents the phylogenetic distances between the used sequences is indeed advantageous when adding closely related sequences. Closely related sequences that are treated independently harm motif detection by inducing multiple local optima as observed for MEME. PG and PS can better handle this problem of local optima as they constrain the search space by prealigning conserved regions and by treating those regions simultaneously. In addition their evolutionary model helps to distinguish conservation due to evolutionary proximity from conservation due to functionality as the prealignment itself is often uninformative [13], [33]. Adding distantly related orthologs usually relieves the problem of the local optima, but often occurs at the cost of the motif quality as motif sites in the distant orthologs are harder to find (less similar to the other motif sites) or the distant orthologs disturbs the prealignment needed for the phylogenetic algorithms. The accuracy of the prealignment seemed in general the major bottleneck for the phylogenetic motif finders. PG in general handles better these difficult to align datasets by combining a local alignment strategy with a more flexible way of assigning motif sites. The different way of treating the prealignment by PG and PS also explains the different behavior of PG and PS towards omitting motif sites in the orthologous direction. For PS we also observed that the use of a weighting scheme in a non-ideal situation (incorrect prealignment and missing motif sites in the distant ortholog) negatively influences the results. This implies that when using PS, the user can better omit distant sequences for which he is not sure that the mode of regulation is still conserved.

Lastly, all used algorithms underperform when searching for motifs in only a set of orthologous sequences. This effect was most pronounced for PS that only retrieved an output when leaving the sequences unaligned and suppressing the use of the phylogenetic model. This failure of PS relates to the fact that the ‘sampling model-update step’ (see Table S1: ‘Algorithm: sampling’) does not sufficiently explores the search space in the absence of coregulated information. PG which uses a different search strategy better explores the search space in the orthologous space.

Having an insight in this relation between the obtained results and the working principles of the algorithms provides developers hints for further improvements. For instance the ease with which a basic algorithm as MEME can be used largely compensates for the slightly higher accuracy that is obtained with the more complex phylogenetic algorithms. Based on our experience we would therefore suggest of using MEME to get a first insight into the data. This will help tuning the parameters of the more complex phylogenetic algorithms that on their turn can further improve the results e.g. by retrieving more ‘true’ and less ‘false positive’ sites. User-friendliness is one of the major issues in determining which algorithm to use. Most of the current phylogenetic algorithms are still in their developmental phase and do not yet provide the same user-friendliness as more settled algorithms such as MEME. Moreover, as the quality of the results of the phylogenetic algorithms heavily depends on the correctness of the prealignments, developing ways to account for phylogenetic relatedness, independent of a prealignment is a future challenge. Care should also be taken when introducing specific ways to model the relation between the orthologous sequences. For instance, for PS the use of the weighting scheme has a very counterintuitive effect when motif sites are missing in the orthologous direction. The development of algorithms that can better cope with phenomena of ‘motif site turnover’ during evolution [34] will hopefully result in more realistic and informative models. Lastly the ensemble strategy of PS definitely is useful, but can be computationally limiting.

## Supporting Information

Figure S1 Figure S1 depicts the phylogenetic trees used to relate the eight Gamma-proteobacterial species and the five Saccharomyces species.
(0.07 MB DOC)

Table S1 Table S1 summarizes the most important characteristics of Phylogibbs (PG) and Phylogenetic sampler (PS).
(0.10 MB DOC)

Table S2 Table S2 explains the composition of the real datasets for the Gamma-proteobacterial and the Saccharomyces species.
(0.06 MB DOC)

Table S3 Table S3 shows the effect of different types of phylogenetic trees on the results of the phylogenetic algorithms (PG and PS) for the Gamma-proteobacterial datasets in the combined coregulation-orthology space.
(0.05 MB DOC)

Table S4 Table S4 contains the Newick formats for all the phylogenetic trees that were used in the tests on the synthetic and real datasets.
(0.04 MB DOC)

Table S5 Table S5 consists of Tables S5 (A, B, C and D) containing the results of PG, PS and MEME in the coregulation space and in the combined coregulation-orthology space for both the synthetic and real datasets.
(0.17 MB DOC)

Table S6 Table S6 shows the effect of leaving out motif sites in both the coregulation space and in the combined coregulation-orthology space for a synthetic dataset containing sites sampled from a high IC motif.
(0.08 MB DOC)

Table S7 Table S7[Supplementary-material pone.0008938.s008] gives the results of the phylogenetic algorithms when using orthologs related through a non star like tree topology for the synthetic data in the combined coregulation-orthology space.
(0.04 MB DOC)

Table S8 Table S8 consists of Tables S8 (A and B) containing the results of PG, PS and MEME in the orthologous space.
(0.15 MB DOC)

Text S1 Text S1 provides additional information on the construction of the synthetic and real datasets.
(0.05 MB DOC)

Text S2 Text S2 provides additional information on the pre-processing of the alignments.
(0.07 MB DOC)

Text S3 Text S3 provides additional information on the parametersettings.
(0.08 MB DOC)
