# Supplementary material for: The Effect of Orthology and Coregulation on Detecting Regulatory Motifs
Source: PLoS One. 2010 Feb 3;5(2):e8938. doi: 10.1371/journal.pone.0008938 (PMC2815771; doi:10.1371/journal.pone.0008938)
Supplement: Figure S1 — depicts the phylogenetic trees used to relate the eight Gamma-proteobacterial species and the five Saccharomyces species. (0.07 MB DOC) [file pone.0008938.s001.doc]

**
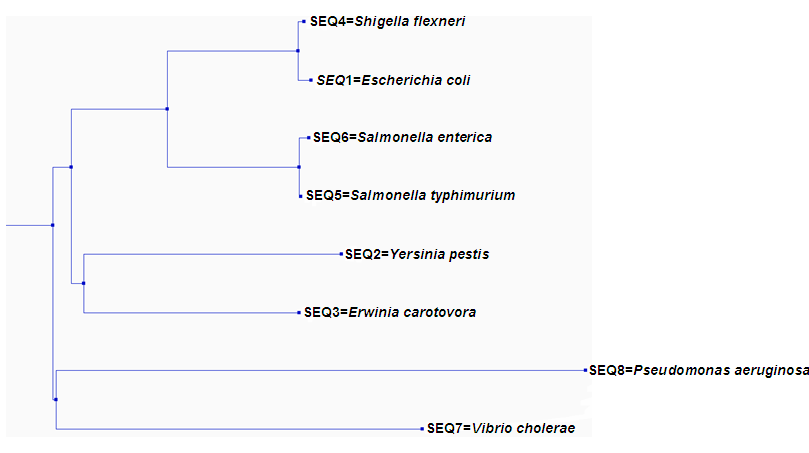
**


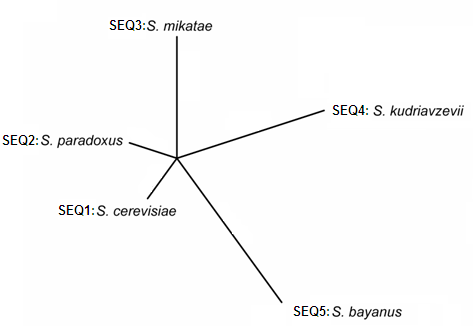


**Figure S1** Phylogenetic trees relating the eight Gamma-proteobacterial species (on top) and the five *Saccharomyces* species (below). Both trees (Newick formats in Table S4) are based on a neutral evolution rate and reflect the phylogenetic relatedness between the intergenic sequences of eight Gamma-proteobacterial species (on top) or five *Saccharomyces* species (below). For the tests on real data for the combined and the orthologous space, we added orthologs with increasing phylogenetic distances to the reference species *E.coli* (in case of the bacterial datasets) or *S. cerevisiae* (in case of the yeast datasets). Subsets of these orthologs (reference species included) used throughout the tests were selected as follows:

**For the Gamma-proteobacteria:**

2 orthologs = SEQ1+SEQ4; 4 orthologs = SEQ1+SEQ4+SEQ5+SEQ6;

6 orthologs = SEQ1+SEQ4+SEQ5+SEQ6+SEQ2+SEQ3;

7 orthologs = SEQ1+SEQ4+SEQ5+SEQ6+SEQ2+SEQ3+SEQ8

and 8 orthologs = SEQ1+SEQ4+SEQ5+SEQ6+SEQ2+SEQ3+SEQ7+SEQ8.

**For the *Saccharomyces* species:**

2 orthologs = SEQ1+SEQ2; 4 orthologs = SEQ1+SEQ2+SEQ3+SEQ4; 5 orthologs = SEQ1+SEQ2+SEQ3+SEQ4+SEQ5.
