# Supplementary material for: The Effect of Orthology and Coregulation on Detecting Regulatory Motifs
Source: PLoS One. 2010 Feb 3;5(2):e8938. doi: 10.1371/journal.pone.0008938 (PMC2815771; doi:10.1371/journal.pone.0008938)
Supplement: Table S1 — summarizes the most important characteristics of Phylogibbs (PG) and Phylogenetic sampler (PS). (0.10 MB DOC) [file pone.0008938.s002.doc]

**Table S1** Summary of the most important characteristics of Phylogibbs (PG) [1–3] and Phylogenetic sampler (PS) [4–6].

| **PG** | **PS** |
| --- | --- |
| **MODEL** | |
| **Input Sequences** | |
| -When used in the coregulation space, input sequences consist of intergenic regions of coregulated genes from one species. When used in the orthologous space, input sequences consist of orthologous intergenic regions that can optionally be prealigned. When used in the combined space, the input sequences consist of intergenic regions of coregulated genes complemented with the orthologs of these genes (that again can be optionally prealigned).  -Both algorithms model the input sequences as generated by a *background model.* Some positions, deviating from the background model are assumed to be binding sites for transcription factors (motif sites). These motif sites are modeled by a *motif model*, more specifically a position specific weight matrix (WM). | |
| **Motif model** | |
| -A WM of dimension (4xw) describes the probability of finding the respective nucleotides A, C, G, T at each position (from 1 to w) in the motif site. Motif sites belonging to a specific transcription factor are described by the same WM.  **-Both algorithms define motif sites differently for:** | |
| **1. Prealigned orthologous sequences** | |
| -Assignment of a set of evolutionary related motif sites conserved across multiple species from the alignment.  Terminology: a *window* (more specific a multi-species window).  - Windows containing gaps can be split up into smaller windows without gaps and less species (see Text S2). PG can work on **subparts** of the alignment, allowing windows to be placed in conserved, well aligned regions as well as in unaligned regions. Therefore prealignments are made by a local alignment tool (Dialign was recommended) that annotates aligned and unaligned regions. | -Assignment of a set of evolutionary related motif sites conserved across **all** species from the alignment.  Terminology: a *block*.  Also used by PS is the term *MASS* defined as a set of aligned orthologs.  -PS works on the alignment as a **whole**. Only sets of motif sites conserved over all species are taken into account, excluding all sets containing gaps. Prealignments for PS are based on a global alignment strategy and PS recommends ClustalW. |
| **2. Sequences from one species and unaligned orthologous sequences** | |
| Assignment of individual independent motif sites (a *window* now consists of one motif site, more specific a single-species window). | Assignment of individual independent motif sites (a *block* now consists of one motif site and a *MASS* is one sequence). |
| **Background model** | |
| Markov model with order n defined by the user. This model gives the probability of each base given the bases on the n previous positions. Model parameters are estimated based on input sequences or based on an external file with intergenic sequences. | Position specific background model. This model gives the probability of each base on each position of the sequence and can be regarded as a zero-order Markov model. The model parameters are estimated based on the input sequences. |

| **Evolutionary model** | | |
| --- | --- | --- |
| -**The model for evolution** used by both algorithms is an *adapted F81 model* [7]:  The adapted F81 model describes the probability$P_{ab}(t)$that nucleotide a is mutated to nucleotide b over a time period t. The model assumes that all sequence positions evolve independently and at equal rates (u) and the probability for fixation of a mutation at position i is proportional to the WM entry of that nucleotide at position i. $P_{ab}(t)=\exp(-ut)\delta_{ab}+(1-\exp(-ut))WM_{b,i}$   With $u$ = substitution rate, $t$ =time, $\delta_{ab}$ is the Kronecker delta function that equals one for a=b and zero for a≠b and $WM_{b,i}$ is the WM entry of nucleotide b for position i (respectively the motif and background WM).  Note that this model is an extension of the F81 model [8] where fixation of a mutation is proportional to the frequency of that nucleotide in the data $\pi_{b}$ (instead of$WM_{b,i}$). | | |
| **-The evolutionary relations between the species in the dataset are modeled by a phylogenetic tree with two properties:**    **1. A set of phylogenetic distances:** The phylogenetic distance between two species is modeled by: | | |
| The p*roximity q* between two species = probability that no substitution took place per site. q~exp(-ut) is used in the above evolutionary model. | The *branch length b* between two species = the expected number of substitutions per site. b~ut is used in the above evolutionary model. | |
| **2. A topology (pattern of branching):** Each branch connects a species/internal node with an internal node/ ancestor. | | |
| PG is only directly applicable to star topology trees where all species are directly descending from one common (unknown) ancestor. | PS is directly applicable to all tree topologies and thus allows for unknown internal nodes in the tree. | |
| **ALGORITHM** | | |
| -Goal: identify the positions of the motif sites hidden in the input sequences.  -Method: explore the space of all possible solutions by MCMC [9] sampling. | | |
| **Sampling** | | |
| -Collapsed Gibbs sampling: sample from a sequence of *posterior distributions* along a set of extensive moves.   1. Start with a random positioning of windows, assigned to different TFs, also called a *configuration C*, based on prior information on the expected number of windows per TF in the data. 2. Construct the set of all possible configurations C’ that differ in one single move from C. A move is e.g. changing the position of one single window or adding a new window. 3. *Scoring*: calculate for each C’ the posterior probability score. 4. Sample a new configuration from this score distribution.   This procedure (one cycle) is repeated for two phases :  - 1) *simulated annealing* [10] where one iterates to configuration C* with the highest posterior probability (=MAP). Instead of sampling from the normal score distribution a parameter β was introduced and sampling is done from a distribution which is proportional to (score)^β^. By slowly increasing β the sampler will freeze into the global optimum C*.  -2) *tracking* where posterior probabilities are assigned to the windows in C*.  -> one initialization is sufficient  -> short running time (minutes/hours) | | - Grouped Gibbs sampling: sample from a sequence of *conditional distributions* along a set of systematic moves.   1. Start with a random positioning of blocks, assigned to different TFs, based on prior information on the expected number of blocks per TF in the data and maximum number of blocks per MASS. 2. Update the motif model based on all the current blocks (Model-update step). 3. Scoring: leave out the blocks for one MASS and calculate for each possible block in this MASS the conditional probability score. 4. First sample the number of blocks for the MASS (recursive algorithm), then sample this number of blocks from the score distribution calculated in step 3 (Site-sampling step). 5. Repeat steps 2 till 4 for each MASS in the dataset   This procedure (one iteration) is repeated for two phases :  -1) *burn-in* *iterations* to converge to an optimum.  -2) *sampling iterations* to keep track of all sampled blocks to construct the solution afterwards.  -> multiple initializations (seeds) recommended to avoid getting trapped in local optimum  -> long running time (hours/days) |
| **Scoring** | | |
| **Score in above step 3**  -The *posterior probability* score of a configuration C is proportional with the probability that all windows in C are drawn from (unknown) motif WMs and that the background sequence is drawn from a known background model.  -The motif WM is assumed to be unknown:  -to compute the probability that a window is drawn from an unknown motif WM, PG will use the conditional probability (this is the probability with a known motif WM) and scan this function over the entire WM space. Mathematically this resumes to solving an integral over all possible WMs, where the prior P(WM) is modeled by a Dirichlet prior distribution.  For windows containing evolutionary related sites: the scoring will include an *evolutionary model* and a phylogenetic tree to describe the probability that orthologous sites are related to a common ancestor site.  -For computational reasons, an approximation is needed to solve the integral. This approximation requires a star topology which makes it possible to directly obtain the joint probability of the evolutionary related nucleotides at the leaves of the tree. All other tree topologies are reduced to collections of star topologies. | **Score in above step 3:**  -The *conditional probability* of one block is proportional with the probability that the block is drawn from a known motif WM divided by the probability that the block is drawn from the known background model.  -The motif WM is assumed to be known :  - update WM before score-computation (above step 2) :   - Sample a new motif WM from a Dirichlet distribution Dir(β+c) where β = vector with pseudocounts for each base and c = vector with sequence weighted counts ^(*)^ for each base across all the blocks. - Accept the new motif WM with a probability proportional to the Metropolis Hastings ratio. This ratio is proportional with how good the new model explains the blocks versus the old model.   ^(*)^ Each orthologous sequence gets a weight based on the phylogenetic tree relating them by using the program Seq.weights.pl. For details on using sequence weights to build a motif WM see [11].  For blocks containing evolutionary related sites: the scoring will include an *evolutionary model* and a phylogenetic tree to describe the probability that orthologous sites are related to a common ancestor site.  -The *Felsenstein tree-likelihood algorithm* [8] is used to handle all tree topologies. It is a recursive algorithm that marginalizes over all the interior nodes of the tree to obtain the joint probability of the nucleotides at the leaves of the tree. | |
| **Solution** | | |
| **Maximum a posteriori (MAP) solution**  The output contains the configuration C* that has the highest posterior probability. It is an optimization based solution. | **Ensemble centroid solution**  The centroid solution (also used in the ‘Gibbs Centroid sampler’ [5]) is a collection of centroid motif sites composed by all the block positions that appear in at least half the sampling iterations over different initializations. | |

| **Posterior probabilities** | |
| --- | --- |
| -During *the tracking phase* PG samples the distribution P(C|S) of all configurations and compares each sampled configuration with the MAP configuration C* to assign posterior probabilities to all windows it reports.  -The posterior probability of a window reports how strong this window is member of, or associated with C*.  -Windows with a posterior probability higher than the chosen *tracking threshold* (T) are reported in the ‘track output’ file. | - A posterior probability is assigned to each centroid site based on the number of times the site or overlapping sites were sampled on the total number of iterations.  -The *align-centroid* option aligns the centroid motif sites for a specific TF to construct a motif WM by using the ‘Gibbs recursive sampler’ [4]. |

Reference List

1. Siddharthan R (2007) Parsing regulatory DNA: general tasks, techniques, and the PhyloGibbs approach. J Biosci 32: 863-870.

2. Siddharthan R, van Nimwegen E (2007) Detecting regulatory sites using PhyloGibbs. Methods Mol Biol 395: 381-402.

3. van Nimwegen E (2007) Finding regulatory elements and regulatory motifs: a general probabilistic framework. BMC Bioinformatics 8 Suppl 6: S4.

4. Thompson W, Rouchka EC, Lawrence CE (2003) Gibbs Recursive Sampler: finding transcription factor binding sites. Nucleic Acids Res 31: 3580-3585.

5. Thompson WA, Newberg LA, Conlan S, McCue LA, Lawrence CE (2007) The Gibbs Centroid Sampler. Nucleic Acids Res 35: W232-W237.

6. Newberg LA, Thompson WA, Conlan S, Smith TM, McCue LA, et al. (2007) A phylogenetic Gibbs sampler that yields centroid solutions for cis-regulatory site prediction. Bioinformatics 23: 1718-1727.

7. Sinha S, van Nimwegen E, Siggia ED (2003) A probabilistic method to detect regulatory modules. Bioinformatics 19 Suppl 1:I292-I301.: I292-I301.

8. Felsenstein J (1981) Evolutionary trees from DNA sequences: a maximum likelihood approach. J Mol Evol 17: 368-376.

9. Liu JS (2001) Monte Carlo Strategies in Scientific Computing. Springer Series in Statistics. 360 p.

10. Kirkpatrick S, Gelatt CD, Jr., Vecchi MP (1983) Optimization by Simulated Annealing. Science 220: 671-680.

11. Newberg LA, McCue LA, Lawrence CE (2005) The relative inefficiency of sequence weights approaches in determining a nucleotide position weight matrix. Stat Appl Genet Mol Biol 4: Article13.
