# Supplementary material for: The Effect of Orthology and Coregulation on Detecting Regulatory Motifs
Source: PLoS One. 2010 Feb 3;5(2):e8938. doi: 10.1371/journal.pone.0008938 (PMC2815771; doi:10.1371/journal.pone.0008938)
Supplement: Table S2 — explains the composition of the real datasets for the Gamma-proteobacterial and the Saccharomyces species. (0.06 MB DOC) [file pone.0008938.s003.doc]

**Table S2** Composition of the real datasets for the Gamma-proteobacterial and the *Saccharomyces* species.

| GAMMA-PROTEOBACTERIA | | | | | | | | | | |
| --- | --- | --- | --- | --- | --- | --- | --- | --- | --- | --- |
| **R** | HIGH IC – LexA | | | | | | | | | |
| **T** | LexA | PolB | RecN | RpsU* | SulA | UvrA* | UvrB | UvrD |  | |
| **M** | 3 | 1 | 2 | 1 | 1 | 1 | 1 | 1 |
| **O** | 8 | 8 | 8 | 8 | 6 | 8 | 8 | 8 |
| **S^[1]^** | / | / | / | / | 7,8 | / | / | / |
| **R** | LOW IC – TyrR | | | | | | | | | |
| **T** | AroF | AroG | AroL | Mtr* | TyrB* | TyrP | TyrR |  | | |
| **M** | 3 | 1 | 5 | 1 | 1 | 2 | 2 |
| **O** | 8 | 5 | 6 | 7 | 7 | 6 | 8 |
| **S^[1]^** | / | 2,7,8 | 7,8 | 7 | 7 | 7,8 | / |
| *SACCHAROMYCES* SPECIES | | | | | | | | | | |
| **R** | HIGH IC - URS1H | | | | | | | | | |
| **T** | AGP1 | SPO16 | REC104 | IME2* | REC114* | MEK1 | HOP1 | MSH5 | MRPL27 | POP4 |
| **M** | 1 | 1 | 1 | 1 | 1 | 2 | 1 | 1 | 1 | 1 |
| **O** | 5 | 5 | 5 | 5 | 5 | 5 | 5 | 5 | 5 | 5 |
| **S^[2]^** | / | / | / | / | / | / | / | / | / | / |
| **R** | LOW IC - RAP1 | | | | | | | | | |
| **T** | HIS4* | RPL11B | ENO1* | OPI3 | AVT4 | BUD22 | RPS2 | YEF3 | SNF4 | RPL5 |
| **M** | 1 | 1 | 1 | 1 | 1 | 1 | 1 | 1 | 1 | 1 |
| **O** | 5 | 5 | 5 | 5 | 5 | 5 | 5 | 5 | 5 | 5 |
| **S^[2]^** | / | / | / | / | / | / | / | / | / | / |

**Rows: R**: the regulator, **T**: the target genes for the regulator used to compose the dataset in the coregulation space in respectively the reference species *E. coli* and *S. cerevisiae*, **M:** number of motif sites for the specific regulator in the selected upstream region of those reference genes, **O:** the total number of orthologs that could be used for each target gene in the orthologous or combined space (the number includes the gene in the reference species itself) and **S:** the species for which no ortholog was retrieved. Species names are represented by the following numbers: ^[1]^ The gamma proteobacteria: *1*=*Escherichia coli*, *2*=*Yersinia pestis*, *3*=*Erwinia carotovora*, *4*=*Shigella flexneri*, *5*=*Salmonella typhimurium*, *6*=*Salmonella enterica*, *7*=*Vibrio cholerae*, *8*=*Pseudomonas aeruginosa*.

^[2]^ For both, URS1H and RAP1, all 10 target genes in the reference species had orthologs in all 4 other *Saccharomyces* species.

Note that the used version of PS can not handle a dataset for which the MASSES (~a prealigned set of orthologs for one gene) contain a different number of orthologs. Therefore, we left out the gene AroG in the TyrR datasets for the tests with PS. We applied this correction also in the coregulation space to better compare results obtained from the combined and orthologous space with those from the coregulation space.

*Target genes in the reference species for respectively LexA, TyrR, URS1H and RAP1: each of those was used together with its respective orthologs for the tests in the orthologous space.
