# Supplementary material for: The Effect of Orthology and Coregulation on Detecting Regulatory Motifs
Source: PLoS One. 2010 Feb 3;5(2):e8938. doi: 10.1371/journal.pone.0008938 (PMC2815771; doi:10.1371/journal.pone.0008938)
Supplement: Table S3 — shows the effect of different types of phylogenetic trees on the results of the phylogenetic algorithms (PG and PS) for the Gamma-proteobacterial datasets in the combined coregulation-orthology space. (0.05 MB DOC) [file pone.0008938.s004.doc]

**Table S3** The effect of different types of phylogenetic trees on the results of the phylogenetic algorithms (PG and PS) for the Gamma-proteobacterial datasets in the combined coregulation-orthology space.

| GAMMA-PROTEOBACTERIA | | | | | | | | |
| --- | --- | --- | --- | --- | --- | --- | --- | --- |
| SETUP | HIGH IC - LexA | | | | LOW IC - TyrR | | | |
| **Results of PG** | | | | | | | | |
| **Tree type** | **R1** | **RR** | **spPPV** | **spSens** | **R1** | **RR** | **spPPV** | **spSens** |
| Neutral | 10 | 100 | 98.6 | 75.5 | 8 | 100 | 96.9 | 67.5 |
| Protein | 2 | 50 | 85.7 | 54.5 | 2 | 0 | / | / |
| Corrected | 10 | 90 | 95.6 | 72.7 | 8 | 75 | 92.8 | 61.1 |
| **Results of PS** | | | | | | | | |
| **Tree type** | **R1** | **RR** | **spPPV** | **spSens** | **R1** | **RR** | **spPPV** | **spSens** |
| Neutral | 10 | 90 | 69 | 42.2 | 10 | 100 | 85 | 36.4 |
| Protein | 0 | / | / | / | 0 | / | / | / |
| Corrected | 10 | 80 | 71.4 | 45.5 | 10 | 100 | 100 | 40 |

**Performance and quality measures: R1**: the number of runs with an output out of the 10 runs on one real dataset, **RR (%)**: Recovery Rate: the percentage of the output (R1) for which the correct motif was retrieved (correct outputs), **spPPV (%)**: species-dependent PPV: the percentage of true sites among the predicted sites for the reference species, averaged over all correct outputs, **spSens (%)**: species-dependent Sens: the percentage of the true sites in the reference species found by the algorithm, averaged over all correct outputs. *E. coli* is the reference species. The dataset of each regulator consists of 8 (LexA) or 7 (TyrR) target genes from the reference species (see Table S2), together with their orthologs in 5 additional species (Figure S1 lists from which species these orthologs were derived). Each reference sequence together with its orthologs was prealigned (6 sequences in total). The phylogenetic relatedness between the orthologous sequences is modeled by one of the three different ‘*tree types’*: a Neutral, a Protein or a Corrected tree (Newick formats in Table S4).

**Additional information on the phylogenetic trees used for the synthetic and real datasets**

The construction of the tree used to model the phylogenetic relatedness between the intergenic sequences of the Gamma-proteobacteria was based on coding sequence alignments. Three different trees were obtained : 1) a tree made by PhyML [1] with as input the alignment of 30 concatenated protein sequences over all eight species (~Protein tree), 2) the branch lengths of the previous tree multiplied by factor 13.5 as described by Newberg *et al.* [2] (~Corrected tree) and 3) a tree based on neutral evolution rates by only taking into account the evolution of the third positions of four fold degenerate codons (~Neutral tree) (kindly provided by Erik Van Nimwegen). As can be seen in Table S3, for both PG and PS, the overall highest recovery rate (RR) was obtained with the tree based on a neutral evolution rate. The tree based on protein alignments did most of the time not result in any output as it overestimates the relatedness between the intergenic sequences. As intergenic sequences are expected to evolve faster than coding sequences, correcting the branch lengths of the protein tree allowed to better approximate the true relatedness between the intergenic sequences and the obtained results became more comparable to those obtained with the neutral tree. In all subsequent analyses, the tree based on the neutral evolution rate was used [3]. Figure S1 shows the neutral evolution tree for both the Gamma-proteobacterial and the *Saccharomyces* species. The Newick formats of all trees, used for tests on synthetic and real datasets, are given in Table S4.

Reference List

1. Guindon S, Gascuel O (2003) A simple, fast, and accurate algorithm to estimate large phylogenies by maximum likelihood. Syst Biol 52: 696-704.

2. Newberg LA, Thompson WA, Conlan S, Smith TM, McCue LA, et al. (2007) A phylogenetic Gibbs sampler that yields centroid solutions for cis-regulatory site prediction. Bioinformatics 23: 1718-1727.

3. Siddharthan R, Siggia ED, van Nimwegen E (2005) PhyloGibbs: a Gibbs sampling motif finder that incorporates phylogeny. PLoS Comput Biol 1: e67.
