# Supplementary material for: The Effect of Orthology and Coregulation on Detecting Regulatory Motifs
Source: PLoS One. 2010 Feb 3;5(2):e8938. doi: 10.1371/journal.pone.0008938 (PMC2815771; doi:10.1371/journal.pone.0008938)
Supplement: Table S4 — contains the Newick formats for all the phylogenetic trees that were used in the tests on the synthetic and real datasets. (0.04 MB DOC) [file pone.0008938.s005.doc]

**Table S4** Newick format for all the phylogenetic trees used in the tests on the synthetic and real data. The distances are given in proximities (q).

| **Synthetic data** | | |
| --- | --- | --- |
| **Topology** | | **Proximities** |
| Star | Equal distances ^[1]^ | (SEQ1:0.80,SEQ2:q,SEQ3:q,SEQ4:q,SEQ5:q); (5orthologs)  (SEQ1:0.80,SEQ2:q,SEQ3:q,SEQ4:q,SEQ5:q, SEQ6:q,SEQ7:q,  SEQ8:q,SEQ9:q,SEQ10:q); (10 orthologs) |
|  | Unequal distances | (SEQ1:0.80,SEQ2:0.90,SEQ3:0.85,SEQ4:0.75,SEQ5:0.20); |
| Tree [6] | | (((SEQ1:0.83,(SEQ2:0.89,SEQ3:0.91):0.84):0.95,  (SEQ4:0.97,SEQ5:0.99):0.82):0.93,SEQ6:0.70); |
| **Real data ^[2]^** | | |
| **Gamma-proteobacteria** | | |
| **Topology** | | **Proximities** |
| Tree | Neutral | ((((SEQ1:0.95,SEQ4:0.98):0.61,(SEQ5:0.99,SEQ6:0.96):0.61):0.70,  (SEQ2:0.38,SEQ3:0.45):0.95):0.94,(SEQ7:0.25,SEQ8:0.14):0.99); |
|  | Protein | ((((SEQ1:0.99,SEQ4:0.99):0.97,(SEQ5:0.99,SEQ6:0.99):0.97):0.94,  (SEQ2:0.92,SEQ3:0.91):0.96):0.79,SEQ7:0.76,SEQ8:0.38); |
|  | Corrected ^[3]^ | ((((SEQ1:0.99,SEQ4:0.92):0.68,(SEQ5:0.99,SEQ6:0.97):0.66):0.43,  (SEQ2:0.32,SEQ3:0.29):0.58):0.04,SEQ7:0.02,SEQ8:0.0); |
| ***Saccharomyces* species** | | |
| **Topology** | | **Proximities** |
| Star | Neutral | (SEQ1:0.80,SEQ2:0.80,SEQ3:0.58,SEQ4:0.50,SEQ5:0.45); |

^[1]^ The value for q varies between 0.90, 0.50 and 0.20 for different tests. For example: q=0.90 describes a phylogenetic tree for very closely related orthologs.

^[2]^ For the real data we replaced the species names by SEQ*numbers.* For the **Gamma-proteobacteria**: *1*=*Escherichia coli*, *2*=*Yersinia pestis*, *3*=*Erwinia carotovora*, *4*=*Shigella flexneri*, *5*=*Salmonella typhimurium*, *6*=*Salmonella enterica*, *7*=*Vibrio cholerae*, *8*=*Pseudomonas aeruginosa*. For the **Saccharomyces species**: *1=S. cerevisiae, 2=S. paradoxus, 3=S. mikatae, 4=S. kudriavzevii, 5=S. bayanus*,

^[3]^ The relation between the proximities for the Protein and the Corrected tree is given by: (proximity of the Corrected tree) = (proximity of the Protein tree)^13.5^ as described in [1]. The proximities are rounded up to two decimals after the comma.

1. Newberg LA, Thompson WA, Conlan S, Smith TM, McCue LA, et al. (2007) A phylogenetic Gibbs sampler that yields centroid solutions for cis-regulatory site prediction. Bioinformatics 23: 1718-1727.
