# Supplementary material for: The Effect of Orthology and Coregulation on Detecting Regulatory Motifs
Source: PLoS One. 2010 Feb 3;5(2):e8938. doi: 10.1371/journal.pone.0008938 (PMC2815771; doi:10.1371/journal.pone.0008938)
Supplement: Table S5 — consists of Tables S5 (A, B, C and D) containing the results of PG, PS and MEME in the coregulation space and in the combined coregulation-orthology space for both the synthetic and real datasets. (0.17 MB DOC) [file pone.0008938.s006.doc]

**Table S5 (A, B, C and D)** Results of PG, PS and MEME in the coregulation space and in the combined coregulation-orthology space.

All data presented in Table S5 (A, B and C), showing the results of the three algorithms on the *synthetic datasets* in the coregulation space and in the combined space are described in the main text. Table S5 (D) shows the results of the three algorithms on the *real datasets* in the coregulation and combined space. Also these results have been described in the main text, except for the results **in the coregulation space.** For those results, a trend similar as for the synthetic datasets in Table S5 (A) was observed, though less pronounced. For both, the Gamma-proteobacterial and yeast datasets, all algorithms retrieved the high information content (IC) motif (LexA or URS1H) with a high RR and motif quality. For a low IC motif (TyrR or RAP1) the quality of the motifs retrieved by all three algorithms, especially by PG and PS, was characterized by a very pronounced drop in sensitivity (Sens). As for the synthetic data in Table S5 (A), PG showed for a low IC motif a weaker performance (R1 and RR) compared to PS and MEME.

**Table S5 A** Results of PG, PS and MEME on synthetic datasets for the **‘star topology with equal distances’** given in proximities (q). The results for the ‘coregulation space’ are given as reference values (*REF*).

| SYNTHETIC DATA | | | | | | | | |
| --- | --- | --- | --- | --- | --- | --- | --- | --- |
| SETUP | HIGH IC | | | | LOW IC | | | |
| **Results of PG** | | | | | | | | |
| **Proximity q** | **D1** | **RR** | **PPV** | **Sens** | **D1** | **RR** | **PPV** | **Sens** |
| *REF: Coregulation space* | *44* | *100* | *99.3* | *88.4* | 75* | 5.3* | 72.2* | 47.5* |
| 0.90 | 88 | 100 | 99.4 | 98 | 30 | 76.7 | 94.8 | 72.2 |
| 0.50 | 99 | 100 | 100 | 98.9 | 74 | 100 | 98.5 | 80.9 |
| 0.20 | 100 | 100 | 98.5 | 76.9 | 77 | 94.8 | 92 | 33.9 |
| 0.20 (unaligned) | 95 | 100 | 96.7 | 91 | 42 | 97.6 | 89.9 | 50 |
| **Results of PS** | | | | | | | | |
| **Proximity q** | **D1** | **RR** | **PPV** | **Sens** | **D1** | **RR** | **PPV** | **Sens** |
| *REF: Coregulation space* | *100* | *100* | *99.4* | *91.9* | *36* | *80.6* | *94.9* | *57.2* |
| 0.90 | 100 | 100 | 99.8 | 98.1 | 32 | 93.8 | 97.6 | 79 |
| 0.50 | 100 | 100 | 100 | 97.9 | 100 | 100 | 99.5 | 90.6 |
| 0.20 | 100 | 100 | 97.9 | 78.7 | 97 | 100 | 92.7 | 55.5 |
| 0.20 (unaligned) | 100 | 100 | 99.3 | 95.8 | 100 | 100 | 92.5 | 46.4 |
| **Results of MEME** | | | | | | | | |
| **Proximity q** | **D1** | **RR** | **PPV** | **Sens** | **D1** | **RR** | **PPV** | **Sens** |
| *REF: Coregulation space* | *100* | *100* | *93.1* | *92.7* | *100* | *34* | *67.4* | *67.4* |
| 0.90 | 100 | 100 | 95.6 | 94.9 | 100 | 67 | 73.2 | 73.0 |
| 0.50 | 100 | 100 | 94.8 | 94.5 | 100 | 100 | 68.8 | 68.8 |
| 0.20 | 100 | 100 | 94.2 | 94.1 | 100 | 100 | 66.9 | 66.8 |

**Performance and quality measures:** **D1**: the number of datasets with an output out of the 100 synthetic datasets, **RR (%)**: Recovery Rate: the percentage of the output (D1) for which the correct motif was retrieved (correct outputs), **PPV (%)**: Positive Predictive Value: the percentage of true sites among the predicted motif sites, averaged over all correct outputs, **Sens (%):** Sensitivity: the percentage of the true sites found by the algorithm, averaged over all correct outputs. * Tracking threshold PG equal to 0.05 (instead of 0.50).

The synthetic datasets contain in ‘*the coregulation space*’ the 10 coregulated sequences from the reference species each of which has one motif site embedded. In the ‘combined space’ the datasets contain 10 orthologous sets, (an orthologous set is defined as one reference gene and its orthologs). Each orthologous set contains 5 orthologous sequences that are related trough a star topology with equal distances (Newick format in Table S4) and contain one embedded motif site per sequence (high IC or low IC). They can be aligned or left unaligned.

Note that in the *coregulation space* true motifs recovered by PG and PS in general exhibit a higher PPV than motifs recovered by MEME, while for the Sens the opposite is true. This is a consequence of the different working regime of the Sens/PPV trade off which in each of the algorithms is being used. For MEME and PS this trade off is fixed and can not be user specified.

**Table S5 B** Results of PG, PS and MEME algorithms on synthetic datasets for a **‘star topology with unequal distances’** (~ four closely related orthologs and one distantly related ortholog).

| SYNTHETIC DATA | | | | | | | | |
| --- | --- | --- | --- | --- | --- | --- | --- | --- |
| SETUP | HIGH IC | | | | LOW IC | | | |
| **Results of PG** | | | | | | | | |
| **Unequal star topology** | **D1** | **RR** | **PPV** | **Sens** | **D1** | **RR** | **PPV** | **Sens** |
| Only 4 orthologs aligned | 91 | 98.9 | 99.7 | 99 | 36 | 72.2 | 94.7 | 71.2 |
| All 5 orthologs aligned | 99 | 100 | 99.7 | 92.7 | 69 | 94.2 | 96.7 | 64.3 |
| Distant ortholog unaligned | 91 | 100 | 98.7 | 96.3 | 44 | 90.9 | 92.6 | 68.6 |
| **Results of PS** | | | | | | | | |
| **Unequal star topology** | **D1** | **RR** | **PPV** | **Sens** | **D1** | **RR** | **PPV** | **Sens** |
| Only 4 orthologs aligned | 100 | 100 | 99.8 | 98 | 59 | 89.8 | 96.2 | 72.6 |
| All 5 orthologs aligned | 100 | 100 | 99.8 | 96.9 | 100 | 100 | 98.8 | 81.9 |
| Distant ortholog unaligned | 100 | 100 | 99.7 | 98.3 | 90 | 96.7 | 96.8 | 67.9 |
| **Results of MEME** | | | | | | | | |
| **Unequal star topology** | **D1** | **RR** | **PPV** | **Sens** | **D1** | **RR** | **PPV** | **Sens** |
| Only 4 orthologs | 100 | 100 | 96.4 | 95.9 | 100 | 84 | 71.5 | 71.3 |
| All 5 orthologs | 100 | 100 | 96.1 | 95.7 | 100 | 93 | 68.4 | 68.1 |

**Performance and quality measures:** idem as in Table S5 A. Each synthetic dataset in the combined space consists of 10 coregulated sequences from the reference species together with their orthologs. Each reference sequence together with its orthologs is referred to as an orthologous set. A synthetic dataset thus consists of 10 orthologous sets. Each orthologous set contains in total 5 orthologs that are related trough a star topology with unequal distances (Newick format in Table S4) and contain one embedded motif site per sequence (high IC or low IC). **Only 4 orthologs aligned:** we search for motifs in a dataset for which the 10 orthologous sets only contain the 4 closest related orthologs that were aligned. **All 5 orthologs aligned:** the 10 orthologous sets contain 5 prealigned orthologous sequences. **Distant ortholog unaligned:** the 10 orthologous sets contain all 5 orthologs, but only the 4 closely related ones are aligned and the most distant ortholog is left unaligned.

**Table S5 C** The species-dependent quality parameters for PG, PS and MEME on results obtained in the combined space with synthetic datasets containing sequences related through a **star topology with unequal distances**. This Table complements Table S5 B, with values for the species-dependent PPV and species-dependent sensitivity.

| SYNTHETIC DATA | | | | | | | | |
| --- | --- | --- | --- | --- | --- | --- | --- | --- |
| SETUP | HIGH IC | | | | LOW IC | | | |
| **Results of PG** | | | | | | | | |
| **Unequal star topology** | **PPV** | **Sens** | **spPPV** | **spSens** | **PPV** | **Sens** | **spPPV** | **spSens** |
| All 5 orthologs aligned | 99.7 | 92.7 | 98.5 | 84.9 | 96.7 | 64.3 | 96.5 | 47.8 |
| Distant ortholog unaligned | 98.7 | 96.3 | 96.9 | 92.4 | 92.6 | 68.6 | 92.9 | 47.8 |
| **Results of PS** | | | | | | | | |
| **Unequal star topology** | **PPV** | **Sens** | **spPPV** | **spSens** | **PPV** | **Sens** | **spPPV** | **spSens** |
| All 5 orthologs aligned | 99.8 | 96.9 | 99.7 | 96.7 | 98.8 | 81.9 | 98.8 | 81.9 |
| Distant ortholog unaligned | 99.7 | 98.3 | 99 | 94.9 | 96.8 | 67.9 | 95.3 | 49.4 |
| **Results of MEME** | | | | | | | | |
| **Unequal star topology** | **PPV** | **Sens** | **spPPV** | **spSens** | **PPV** | **Sens** | **spPPV** | **spSens** |
| All 5 orthologs | 96.1 | 95.7 | 98 | 92.2 | 68.4 | 68.1 | 73 | 54.5 |

**Performance and quality measures**: idem as in Table S5 A, except for **spPPV (%)**: species-dependent PPV: the percentage of true sites among the predicted sites for the reference species, averaged over all correct outputs, **spSens (%)**: species-dependent Sens: the percentage of the true sites in the reference species found by the algorithm, averaged over all correct outputs. For this specific case the reference species equals the distantly related species (proximity 0.20).

Each synthetic dataset consists of 10 coregulated genes in the reference species together with their orthologs, thus containing 10 orthologous sets. Each orthologous set contains 5 orthologous sequences that are related trough a star topology with unequal distances (Newick format in Table S4) and contain one embedded motif site per sequence (high IC or low IC). ). **All 5 orthologs aligned:** the 10 orthologous sets contain 5 prealigned orthologous sequences. **Distant ortholog unaligned:** the 10 orthologous sets contain all 5 orthologs, but only the 4 closely related ones are aligned and the most distant ortholog is left unaligned.

**Table S5 D Results of PG, PS and MEME on real datasets (Gamma-proteobacterial and *Saccharomyces* species) for motif detection in the ‘combined coregulation-orthology space’. Results for the ‘coregulation space’ are given as reference values (*REF*).**

| GAMMA-PROTEOBACTERIA | | | | | | | | |
| --- | --- | --- | --- | --- | --- | --- | --- | --- |
| SETUP | HIGH IC - LexA | | | | LOW IC – TyrR | | | |
| **Results of PG** | | | | | | | | |
| **# orthologs** | **R1** | **RR** | **spPPV** | **spSens** | **R1** | **RR** | **spPPV** | **spSens** |
| *REF: coregulation space* | *10* | *100* | *98* | *81.8* | *8* | *100* | *92* | *58.3* |
| 2 | 10 | 100 | 94 | 81.8 | 7 | 100 | 91.6 | 61.9 |
| 4 | 10 | 100 | 89 | 74.5 | 10 | 100 | 91.9 | 67.3 |
| 6 | 10 | 100 | 98.6 | 75.5 | 8 | 100 | 96.9 | 67.5 |
| 6 (unaligned) | 10 | 100 | 87.9 | 81.8 | 10 | 50 | 95.3 | 62.7 |
| **Results of PS** | | | | | | | | |
| **# orthologs** | **R1** | **RR** | **spPPV** | **spSens** | **R1** | **RR** | **spPPV** | **spSens** |
| *REF: coregulation space* | *10* | *100* | *100* | *81.8* | *10* | *100* | *100* | *57.1* |
| 2 | 10 | 100 | 96.7 | 79.1 | 10 | 100 | 100 | 64.3 |
| 4 | 10 | 100 | 90 | 71.8 | 10 | 100 | 98.9 | 63.6 |
| 6 | 10 | 90 | 69 | 42.4 | 10 | 100 | 85 | 36.4 |
| 6 (unaligned) | 10 | 100 | 79.8 | 84.5 | 10 | 100 | 100 | 61.4 |
| **Results of MEME** | | | | | | | | |
| **# orthologs** | **R1** | **RR** | **spPPV** | **spSens** | **R1** | **RR** | **spPPV** | **spSens** |
| *REF: coregulation space* | *10* | *100* | *90.9* | *90.9* | *10* | *100* | *73.3* | *73.3* |
| 2 | 10 | 100 | 90.9 | 90.9 | 10 | 100 | 80 | 80 |
| 4 | 10 | 100 | 90.9 | 90.9 | 10 | 100 | 84.6 | 73.3 |
| 6 | 10 | 100 | 100 | 90.9 | 10 | 100 | 85.7 | 80 |
| *SACCHAROMYCES* SPECIES | | | | | | | | |
| SETUP | HIGH IC – URS1H | | | | LOW IC – RAP1 | | | |
| **Results of PG** | | | | | | | | |
| **# orthologs** | **R1** | **RR** | **spPPV** | **spSens** | **R1** | **RR** | **spPPV** | **spSens** |
| *REF: coregulation space* | *10* | *100* | *93.3* | *98.2* | *9** | *22.2** | *87.5** | *20** |
| 2 | 9 | 100 | 96.3 | 99 | 7* | 28.6* | 100* | 10* |
| 4 | 5 | 100 | 100 | 100 | 5* | 40* | 70.3* | 90* |
| 5 | 5 | 100 | 96 | 80 | 4* | 75* | 67.4* | 93.3* |
| 5 (unaligned) | 2 | 50 | 100 | 100 | 8* | 37.5* | 79.1* | 100* |
| **Results of PS** | | | | | | | | |
| **# orthologs** | **R1** | **RR** | **spPPV** | **spSens** | **R1** | **RR** | **spPPV** | **spSens** |
| *REF: coregulation spacey* | *10* | *100* | *92.5* | *100* | *10* | *100* | *87.6* | *71* |
| 2 | 10 | 100 | 91.7 | 100 | 10 | 100 | 75 | 60 |
| 4 | 10 | 100 | 83.9 | 81.8 | 10 | 100 | 90 | 90 |
| 5 | 10 | 100 | 84 | 75.5 | 10 | 100 | 88.8 | 79 |
| 5 (unaligned) | 10 | 100 | 91.7 | 100 | 10 | 100 | 87.6 | 71 |
| **Results of MEME** | | | | | | | | |
| **# orthologs** | **R1** | **RR** | **spPPV** | **spSens** | **R1** | **RR** | **spPPV** | **spSens** |
| *REF: coregulation space* | *10* | *100* | *100* | *100* | *10* | *100* | *90* | *90* |
| 2 | 10 | 100 | 100 | 100 | 10 | 100 | 81.8 | 90 |
| 4 | 10 | 100 | 100 | 100 | 10 | 100 | 81.8 | 90 |
| 5 | 10 | 100 | 100 | 100 | 10 | 100 | 81.8 | 90 |

**Performance and quality measures: R1**: the number of runs with an output out of the 10 runs on one real dataset, **RR (%)**: Recovery Rate: the percentage of the output (R1) for which the correct motif was retrieved (correct outputs), **spPPV (%)**: species-dependent PPV: the percentage of true sites among the predicted sites for the reference species, averaged over all correct outputs, **spSens (%)**: species-dependent Sens: the percentage of the true sites in the reference species found by the algorithm, averaged over all correct outputs. The reference species equals *E. coli* (bacterial data) or *S. cerevisiae* (yeast data).

**Gamma-proteobacteria:** The dataset of each regulator consists of 8 (LexA) or 7 (TyrR) target genes from the reference species (Table S2), together with their orthologs selected from additional species. In total we have “*# orthologs*” orthologs per gene (the ortholog of the reference species included), all prealigned or all left unaligned, related trough the neutral tree for the Gamma-proteobacteria. ***Saccharomyces* species:** The dataset of each regulator (URS1H, RAP1) consists of 10 target genes from the reference species (Table S2), together with their orthologs select from additional species. In total we have “*# orthologs*” orthologs per gene (the ortholog of the reference species included), all prealigned or all unaligned, related trough the neutral tree for the *Saccharomyces* species. Table S4 shows the Newick formats of both trees and Figure S1 lists which species are used for each ‘*# orthologs’* for both the bacterial and yeast species. * Tracking threshold PG equal to 0.05 (instead of 0.50).
