# Supplementary material for: The Effect of Orthology and Coregulation on Detecting Regulatory Motifs
Source: PLoS One. 2010 Feb 3;5(2):e8938. doi: 10.1371/journal.pone.0008938 (PMC2815771; doi:10.1371/journal.pone.0008938)
Supplement: Table S6 — shows the effect of leaving out motif sites in both the coregulation space and in the combined coregulation-orthology space for a synthetic dataset containing sites sampled from a high IC motif. (0.08 MB DOC) [file pone.0008938.s007.doc]

**Table S6** The effect of leaving out motif sites in both the ‘coregulation space’ (on top) and in the ‘combined coregulation-orthology space’ (below). Results are displayed for a synthetic dataset containing sites sampled from a high IC motif.

| SYNTHETIC DATA | | | | | |
| --- | --- | --- | --- | --- | --- |
| **COREGULATION SPACE** | SETUP | HIGH IC | | | |
|  | **Results of PG** | | | | |
|  | **Number of motif sites/gene** | **D1** | **RR** | **PPV** | **Sens** |
|  | *Ref: 10(1,1,1,1,1,1,1,1,1,1) ^[a]^* | *44* | *100* | *99.3* | *88.4* |
|  | 10(1,1,1,1,1,1,1,1,0,0) ^[b]^ | 29 | 100 | 96.9 | 87.1 |
|  | **Results of PS** | | | | |
|  | **Number of motif sites/gene** | **D1** | **RR** | **PPV** | **Sens** |
|  | *Ref: 10(1,1,1,1,1,1,1,1,1,1) ^[a]^* | *100* | *100* | *99.4* | *91.9* |
|  | 10(1,1,1,1,1,1,1,1,0,0) ^[b]^ | 100 | 100 | 96.9 | 92.4 |
|  | **Results of MEME** | | | | |
|  | **Number of motif sites/gene** | **D1** | **RR** | **PPV** | **Sens** |
|  | *Ref: 10(1,1,1,1,1,1,1,1,1,1) ^[a]^* | *100* | *100* | *93.1* | *92.7* |
|  | 10(1,1,1,1,1,1,1,1,0,0) ^[b]^ | 100 | 99 | 77.7 | 97 |
| **COMBINED COREGULATION-ORTHOLOGY SPACE** | SETUP | HIGH IC | | | |
|  | **Results of PG** | | | | |
|  | **Number of motif sites/gene, ortholog** | **D1** | **RR** | **PPV** | **Sens** |
|  | *Ref: all genes and all orthologs contain the motif site ^[a]^* | *99* | *100* | *99.7* | *92.7* |
|  | Motif site absent in distant ortholog (q=0.20) for all genes ^[b]^ | 76 | 100 | 97.6 | 91.4 |
|  | Motif site absent in close ortholog (q=0.75) for all genes ^[b]^ | 77 | 88.3 | 86.2 | 63.3 |
|  | Motif site absent in all orthologs for two out of ten genes ^[b]^ | 97 | 100 | 98.2 | 96.6 |
|  | **Results of PS** | | | | |
|  | **Number of motif sites/gene, ortholog** | **D1** | **RR** | **PPV** | **Sens** |
|  | *Ref: all genes and all orthologs contain the motif site ^[a]^* | *100* | *100* | *99.8* | *96.9* |
|  | Motif site absent in distant ortholog (q=0.20) for all genes ^[b]^ | 18 | 83.3 | 78.1 | 62.7 |
|  | Motif site absent in close ortholog (q=0.75) for all genes ^[b]^ | 99 | 99 | 79.3 | 88.3 |
|  | Motif site absent in all orthologs for two out of ten genes ^[b]^ | 100 | 100 | 99.5 | 96.5 |
|  | **Results of MEME** | | | | |
|  | **Number of motif sites/gene, ortholog** | **D1** | **RR** | **PPV** | **Sens** |
|  | *Ref: all genes and all orthologs contain the motif site ^[a]^* | *100* | *100* | *96.1* | *95.7* |
|  | Motif site absent in distant ortholog (q=0.20) for all genes ^[b]^ | 100 | 100 | 79.0 | 98.6 |
|  | Motif site absent in close ortholog (q=0.75) for all genes ^[b]^ | 100 | 100 | 77.7 | 96.8 |
|  | Motif site absent in all orthologs for two out of ten genes ^[b]^ | 100 | 100 | 77.9 | 97.1 |

**Performance and quality measures: D1**: the number of datasets with an output out of the 100 synthetic datasets, **RR (%)**: Recovery Rate: the percentage of the output (D1) for which the correct motif was retrieved (correct outputs), **PPV (%)**: Positive Predictive Value: the percentage of true sites among the predicted motif sites, averaged over all correct outputs, **Sens (%):** Sensitivity: the percentage of the true sites found by the algorithm, averaged over all correct outputs. **Number of motif sites/gene** [e.g. 10(1,1,1,1,1,1,1,1,0,0)]: the number before the brackets represents the total number of coregulated sequences for the reference species present in the dataset. The numbers between brackets indicate gene per gene the number of embedded motif sites in the gene’s intergenic sequences. For ‘*the coregulation space*’ each synthetic dataset consists of 10 sequences from the reference species with one/zero motif site embedded. For the ‘*combined space*’ each synthetic dataset contains 10 orthologous sets (an orthologous set is defined as one reference sequence and its orthologs). Each orthologous set consists in total of 5 prealigned orthologs, related trough **a star topology with unequal distances** (Newick format in Table S4 on the Supplementary) and one/zero embedded motif site per sequence. Note that for MEME the orthologs are always unaligned.

^[a]^ Ask the motif detection algorithm to search for the exact number of motif sites present in the dataset.

^[b]^ Ask the motif detection algorithm to search for 1 motif site per sequence, hereby slightly overestimating the number of motif sites present in the dataset.

As mentioned in the main text, the prealignment of the orthologous sequences plays a role in how missing motif sites affect the motif detection results in the combined space, more specifically when motif sites were omitted in all the sequences of one of the species that were added as additional orthologous information to the coregulated gene set of the reference species. Here we provide a more in depth explanation why this is the case**.**

The performance of PG was most deteriorated if the motif sites were omitted in the sequences from a closely related species while for PS the performance was most affected if the motif sites were absent in the sequences of a distantly related species.

The local alignment strategy used in combination with PG will leave the distant orthologs that shows low similarity with the closely related orthologs unaligned. PG thus will only take into account the alignment of the closely related orthologs and will treat the distant orthologs as independent, unaligned sequences. However, when motif sites are missing in the sequences of a closely related species, this might interfere with finding the correct alignment of the orthologous motif sites present in the other closely related sequences. If so, these motif sites will not longer be captured in the same window and this will result in a decrease in sensitivity of the retrieved motifs. Misalignment will also increase the chance to capture a false positive site in the window, resulting in a decrease of the PPV (see Table S6). The effect of a false positive motif site in a window is dependent on the phylogenetic distance of the ortholog for which this site was retrieved: a window will be more penalized during scoring when containing a false, non conserved site present in a closely related ortholog than when present in a distantly related one. This explains why PG is more sensitive to the presence of noisy sequences in closely related species.

For PS only the regions that are gaplessly aligned over all species in the alignment are considered as potential motif sites (blocks). A missing motif site will result in rejection of a block or it will be replaced by a false positive motif site. This last effect can be observed in the Table S6_species_specific, by comparing the overall PPV (over all species) to the species-dependent PPV (= the PPV in the reference species that has a proximity of 0.80): the overall PPV is 80% of the species-dependent PPV, indicating that one site in a block of five motif sites is indeed a false positive one. Moreover, the absence of motif sites in a distant ortholog interferes more in obtaining a good global alignment than when absent in a close ortholog that aligns any way well over the remainder of its sequence. This explains why PS can cope better with the absence of motif sites in closely related orthologs than in distant orthologs

**Table S6_species_specific** Species-dependent motif quality parameters for the results obtained by PS in the ‘combined coregulation-orthology space’ when leaving out motif sites. Results are displayed for a synthetic dataset containing sites sampled from a high IC motif.

| **Unequal star topology with** | **PPV** | **Sens** | **spPPV** | **spSens** |
| --- | --- | --- | --- | --- |
| Motif site absent in distant ortholog (q=0.20) for all genes ^[b]^ | 78.1 | 62.7 | 97.7 | 62.7 |
| Motif site absent in close ortholog (q=0.75) for all genes ^[b]^ | 79.3 | 88.3 | 99.2 | 88.4 |

**Performance and quality measures:** idem as in Table S6 except for the spPPV (=species-dependent PPV) and spSens (=species-dependent Sensitivity), both measured for the reference species (q=0.80).

Each synthetic dataset contains 10 orthologous sets (an orthologous set is defined as one reference sequence and its orthologs). Each orthologous set consists in total of 5 prealigned orthologs, related trough **a star topology with unequal distances** (Newick format in Table S4 on the Supplementary) and one/zero embedded motif site per sequence. ^[b]^ Ask the motif detection algorithm to search for 1 motif site per sequence, hereby slightly overestimating the number of motif sites present in the dataset.
