# Supplementary material for: The Effect of Orthology and Coregulation on Detecting Regulatory Motifs
Source: PLoS One. 2010 Feb 3;5(2):e8938. doi: 10.1371/journal.pone.0008938 (PMC2815771; doi:10.1371/journal.pone.0008938)
Supplement: Table S7 — gives the results of the phylogenetic algorithms when using orthologs related through a non star like tree topology for the synthetic data in the combined coregulation-orthology space. (0.04 MB DOC) [file pone.0008938.s008.doc]

**Table S7** Results of the phylogenetic algorithms when using orthologs related through a **non star like topology** for the synthetic datasets in the combined coregulation-orthology space.

| SYNTHETIC DATA | | | | | | | | |
| --- | --- | --- | --- | --- | --- | --- | --- | --- |
| SETUP | HIGH IC | | | | LOW IC | | | |
| **Results of PG** | | | | | | | | |
| **Number of motif sites/gene** | **D1** | **RR** | **PPV** | **Sens** | **D1** | **RR** | **PPV** | **Sens** |
| 10(1,1,1,1,1,1,1,1,1,1) | 97 | 100 | 99.8 | 99.8 | 63 | 90.5 | 95.4 | 78.7 |
| **Results of PS** | | | | | | | | |
| **Number of motif sites/gene** | **D1** | **RR** | **PPV** | **Sens** | **D1** | **RR** | **PPV** | **Sens** |
| 10(1,1,1,1,1,1,1,1,1,1) | 100 | 100 | 99.8 | 99.5 | 83 | 95.2 | 98.6 | 80.1 |

**Performance and quality measures: D1**: the number of datasets with an output out of the 100 synthetic datasets, **RR (%)**: Recovery Rate: the percentage of the output (D1) for which the correct motif was retrieved (correct outputs), **PPV (%)**: Positive Predictive Value: the percentage of true sites among the predicted motif sites, averaged over all correct outputs, **Sens (%):** Sensitivity: the percentage of the true sites found by the algorithm, averaged over all correct outputs. Each synthetic dataset consists of 10 coregulated genes in the reference species together with their orthologs, thus containing 10 orthologous sets. Each orthologous set contains 6 prealigned orthologous sequences that are related trough a non star like topology (Newick format in Table S4) and contain one embedded motif site per sequence (high IC or low IC).

The effect of using orthologs related through a non star like topology on the accuracy of the phylogenetic algorithms

An intrinsic property of PG is that it can only handle star topologies directly during the score calculation of phylogenetically related motif sites as a result of the way it calculates these posterior probability scores (Table S1: ‘Scoring’). To calculate the posterior probabilities the algorithm solves the integral over all possible motif WMs. When an evolutionary model is included, this integral is solved by making an approximation that requires a star topology tree. Any other topology, deviating from a star topology needs to be converted to a collection of star topologies first. PS on the contrary can cope directly with different topologies. It uses a conditional probability for which the motif WM is known and no integration is needed. Moreover, it uses the Felsenstein tree-likelihood algorithm where internal nodes are allowed.

To test whether this intrinsic difference between both algorithms in treating topologies deviating from a star topology has an effect on the performance, we ran both algorithms on synthetic datasets in the combined coregulation-orthology space that exhibited a non star like topology (created by using the phylogenetic tree described in Newberg *et al.* [1] ). Each dataset contains 10 coregulated reference genes, each supplemented with 5 additional orthologs. The evolutionary distances are sufficiently close to guarantee that the intergenic sequences can reliably be aligned. Results are shown in Table S7.

These results suggest that for this non star like topology PS seemingly outperforms PG. Motif detection resulted in more datasets with an output, a slightly higher recovery rate (RR) and a slightly higher quality for the datasets with a correct output, given by the values of the PPV and sensitivity. However, this better performance of PS over PG was also seen for star topologies in the combined coregulation-orthology space (see Table S5 A and B). So it might be a general tendency observed for all topologies and does not really prove that PS is less sensitive than PG in handling topologies different from a star. This observation was also confirmed by the results on the real datasets which have a non star like topology and for which both algorithms showed comparable results.

Conclusively, using a topology different from a star does not result in striking differences between PG and PS in retrieving the true motifs.

Reference List

1. Newberg LA, Thompson WA, Conlan S, Smith TM, McCue LA, et al. (2007) A phylogenetic Gibbs sampler that yields centroid solutions for cis-regulatory site prediction. Bioinformatics 23: 1718-1727.
