# Supplementary material for: The Effect of Orthology and Coregulation on Detecting Regulatory Motifs
Source: PLoS One. 2010 Feb 3;5(2):e8938. doi: 10.1371/journal.pone.0008938 (PMC2815771; doi:10.1371/journal.pone.0008938)
Supplement: Table S8 — consists of Tables S8 (A and B) containing the results of PG, PS and MEME in the orthologous space. (0.15 MB DOC) [file pone.0008938.s009.doc]

**Table S8 (A and B)** give the results of PG, PS and MEME in the orthologous space.

Table S8 (A) shows the results of the three algorithms on the *synthetic datasets* in the orthologous space and Table S8 (B) shows the results of the three algorithms on the *real datasets* in the orthologous space. These results were all described in the main text.

**Table S8 A** Results of PG, PS and MEME on synthetic datasets in the orthologous space.

| SYNTHETIC DATA | | | | | | | | |
| --- | --- | --- | --- | --- | --- | --- | --- | --- |
| SETUP | HIGH IC | | | | LOW IC | | | |
| **Results of PG** | | | | | | | | |
| **Topology - # orthologs** | **D1** | **RR** | **PPV** | **Sens** | **D1** | **RR** | **PPV** | **Sens** |
| EST (0.50) - 5 | 66 | 100 | 100 | 100 | 6 | 100 | 100 | 100 |
| EST (0.50) - 10 | 100 | 100 | 100 | 100 | 88 | 98.9 | 100 | 97.1 |
| EST (0.50) - 10 (unaligned) | 62 | 100 | 99.4 | 93.5 | 20 | 85 | 98.9 | 73.5 |
| EST (0.90) - 5 | 60* | 38.3* | 100* | 100* | 45* | 8.89* | 100* | 100* |
| EST (0.90) - 10 | 97* | 75.3* | 100* | 100* | 87* | 27.6* | 100* | 100* |
| EST (0.90) – 10 (unaligned) | 96* | 64.6* | 97.5* | 100* | 89* | 34.8* | 96* | 99.7* |
| UEST- 4 | 77* | 48.1* | 100* | 100* | 67* | 25.4* | 100* | 100* |
| UEST - 5 (+distant) | 94* | 83* | 99.7* | 99.7* | 66* | 51.5* | 98.2* | 98.2* |
| **Results of PS** | | | | | | | | |
| **Topology - # orthologs** | **D1** | **RR** | **PPV** | **Sens** | **D1** | **RR** | **PPV** | **Sens** |
| EST (0.50) - 5 | 0 | / | / | / | 0 | / | / | / |
| EST (0.50) - 10 | 7 | 100 | 100 | 100 | 2 | 100 | 100 | 100 |
| EST (0.50) - 10 (unaligned) | 100 | 100 | 100 | 94.4 | 79 | 94.9 | 99.4 | 66.3 |
| EST (0.90) – all settings | 0 | / | / | / | 0 | / | / | / |
| UEST – all settings | 0 | / | / | / | 0 | / | / | / |
| **Results of MEME** | | | | | | | | |
| **Topology - # orthologs** | **D1** | **RR** | **PPV** | **Sens** | **D1** | **RR** | **PPV** | **Sens** |
| EST (0.50) - 5 | 100 | 98 | 96.7 | 96.7 | 100 | 48 | 86.6 | 86.6 |
| EST (0.50) - 10 | 100 | 100 | 97.4 | 97.4 | 100 | 75 | 82.9 | 82.9 |
| EST (0.90) - 5 | 100 | 17 | 94.1 | 94.1 | 100 | 6 | 93.3 | 93.3 |
| EST (0.90) - 10 | 100 | 59 | 90.2 | 90.2 | 100 | 20 | 83.5 | 83.5 |
| UEST- 4 | 100 | 38 | 98.7 | 98.7 | 99 | 18.2 | 90.3 | 90.3 |
| UEST - 5 (+distant) | 100 | 64 | 93.4 | 93.4 | 100 | 18 | 82.2 | 82.2 |

**Performance and quality measures:** **D1**: the number of datasets with an output out of the 100 synthetic datasets, **RR (%)**: Recovery Rate: the percentage of the output (D1) for which the correct motif was retrieved (correct outputs), **PPV (%)**: Positive Predictive Value: the percentage of true sites among the predicted motif sites, averaged over all correct outputs, **Sens (%):** Sensitivity: the percentage of the true sites found by the algorithm, averaged over all correct outputs. Each synthetic dataset contains 1 single orthologous set consisting of ‘*# orthologs’* orthologs (i.e. one sequence of the reference species together with all its orthologs), either all being prealigned or all left unaligned, related trough a ‘*Topology*’ topology. EST = Equal Star Topology with proximity q equal to 0.50 or 0.90 and UEST = Unequal Star Topology with four closely related orthologs and one distantly related ortholog. For the Newick formats of the equal and unequal star topology see Table S4. * Tracking threshold PG equal to 0.05 (instead of 0.50). Note that for MEME all orthologs are unaligned.

**Table S8 B** Results of PG, PS and MEME on real datasets (**Gamma-proteobacterial** and ***Saccharomyces* species**) in the orthologous space.

| GAMMA-PROTEOBACTERIA | | | | | | | | | |
| --- | --- | --- | --- | --- | --- | --- | --- | --- | --- |
| SETUP | | HIGH IC - LexA | | | | LOW IC - TyrR | | | |
| **Results of PG** | | | | | | | | | |
|  | **# orthologs** | **R1** | **RR** | **spPPV** | **spSens** | **R1** | **RR** | **spPPV** | **spSens** |
| **G 1** | 6 | 10 | 0 | / | / |  | | | |
|  | 8(LexA)/7(TyrR) | 10 | 0 | / | / | 10* | 0* | /* | /* |
|  | 8/7 (unaligned) | 7 | 0 | / | / | 7* | 14.3* | 50* | 100* |
| **G 2** | 6 | 10 | 100 | 100 | 100 |  | | | |
|  | 8(LexA)/7(TyrR) | 10 | 100 | 100 | 100 | 10* | 100* | 100* | 100* |
|  | 8/7 (unaligned) | 5 | 40 | 100 | 100 | 9* | 88.9* | 49.4* | 100* |
| **Results of PS** | | | | | | | | | |
|  | **# orthologs** | **R1** | **RR** | **spPPV** | **spSens** | **R1** | **RR** | **spPPV** | **spSens** |
| **G1** | 6 | 3 | 0 | / | / |  | | | |
|  | 8(LexA)/7(TyrR) | 0 | / | / | / | 0 | / | / | / |
|  | 8/7 (unaligned) | 2 | 0 | / | / | 8 | / | / | / |
| **G2** | 6 | 0 | / | / | / |  | | | |
|  | 8(LexA)/7(TyrR) | 0 | / | / | / | 0 | / | / | / |
|  | 8/7 (unaligned) | 1 | 100 | 100 | 100 | 8 | / | / | / |
| **Results of MEME** | | | | | | | | | |
|  | **# orthologs** | **R1** | **RR** | **spPPV** | **spSens** | **R1** | **RR** | **spPPV** | **spSens** |
| **G1** | 6 | 10 | 0 | / | / |  |  |  |  |
|  | 8(LexA)/7(TyrR) | 10 | 0 | / | / | 10 | 0 | / | / |
| **G2** | 6 | 10 | 100 | 100 | 100 |  |  |  |  |
|  | 8(LexA)/7(TyrR) | 10 | 100 | 100 | 100 | 10 | 100 | 50 | 100 |
| ***SACCHAROMYCES* SPECIES** | | | | | | | | | |
| SETUP | | HIGH IC – URS1H | | | | LOW IC – RAP1 | | | |
| **Results of PG** | | | | | | | | | |
|  | **# orthologs** | **R1** | **RR** | **spPPV** | **spSens** | **R1** | **RR** | **spPPV** | **spSens** |
| **G1** | 4 | 0* | /* | /* | /* | 10* | 0* | /* | /* |
|  | 5 | 0* | /* | /* | /* | 10* | 0* | /* | /* |
|  | 5 (unaligned) | 4* | 0* | /* | /* | 9* | 88.9* | 100* | 100* |
| **G2** | 4 | 10* | 100* | 100* | 100* | 9* | 0* | /* | /* |
|  | 5 | 10* | 100* | 100* | 100* | 10* | 0* | /* | /* |
|  | 5 (unaligned) | 10* | 100* | 100* | 100* | 10* | 0* | /* | /* |
| **Results of PS** | | | | | | | | | |
|  | **# orthologs** | **R1** | **RR** | **spPPV** | **spSens** | **R1** | **RR** | **spPPV** | **spSens** |
| **G1** | 4 | 0 | / | / | / | 0 | / | / | / |
|  | 5 | 0 | / | / | / | 4 | 0 | / | / |
|  | 5 (unaligned) | 0 | / | / | / | 4 | 50 | 100 | 100 |
| **G2** | 4 | 0 | / | / | / | 0 | / | / | / |
|  | 5 | 0 | / | / | / | 0 | / | / | / |
|  | 5 (unaligned) | 4 | 25 | 100 | 100 | 0 | / | / | / |

| **Results of MEME** | | | | | | | | | |
| --- | --- | --- | --- | --- | --- | --- | --- | --- | --- |
|  | **# orthologs** | **R1** | **RR** | **spPPV** | **spSens** | **R1** | **RR** | **spPPV** | **spSens** |
| **G1** | 4 | 10 | 0 | / | / | 10 | 100 | 100 | 100 |
|  | 5 | 10 | 0 | / | / | 10 | 100 | 100 | 100 |
| **G2** | 4 | 10 | 100 | 100 | 100 | 10 | 0 | / | / |
|  | 5 | 10 | 100 | 100 | 100 | 10 | 0 | / | / |

**Performance and quality measures:** **R1**: the number of runs with an output out of the 10 runs on one real dataset, **RR (%)**: Recovery Rate: the percentage of the output (R1) for which the correct motif was retrieved (correct outputs), **spPPV (%)**: species-dependent PPV: the percentage of true sites among the predicted sites for the reference species, averaged over all correct outputs, **spSens (%)**: species-dependent Sens: the percentage of the true sites in the reference species found by the algorithm, averaged over all correct outputs. The reference species equals *E. coli* (bacterial data) or *S. cerevisiae* (yeast data).

Each real dataset contains one single orthologous set consisting of ‘*# orthologs’* orthologs (i.e. one sequence of the reference species together with all its orthologs), either all being prealigned or all left unaligned, related trough a neutral species tree (Newick formats for the Gamma-proteobacterial or Saccharomyces species trees in Table S4). Figure S1 lists which species were used for each *‘# orthologs’* for both the bacterial and yeast species.

We generated for each regulator two test sets. **Gamma-proteobacteria:** for LexA we selected respectively the targets G1=RpsU and G2=UvrA and for TyrR the targets G1=Mtr or G2=TyrB, each time complemented with their respective orthologs. For TyrR targets, orthologs were only retrieved for 7 out of the 8 Gamma-proteobacterial species. ***Saccharomyces* species:** for URS1H we selected the targets G1= IME2 or G2= REC114 and for the regulator RAP1 the targets G1= HIS4 or G2= ENO1, each time complemented with their respective orthologs. Targets in the reference species contained exactly one motif site for the regulator in their intergenic region.

* Tracking threshold PG equal to 0.05 (instead of 0.50). For MEME all orthologs are unaligned.

Note that for the real data in the orthologous space it is hard to judge on results with a RR=0%, as this might both refer to the detection of a false positive motif or the detection of a true positive different from the annotated motif (so the presence of a second more strong local optimum).
