# Supplementary material for: The Effect of Orthology and Coregulation on Detecting Regulatory Motifs
Source: PLoS One. 2010 Feb 3;5(2):e8938. doi: 10.1371/journal.pone.0008938 (PMC2815771; doi:10.1371/journal.pone.0008938)
Supplement: Text S1 — provides additional information on the construction of the synthetic and real datasets. (0.05 MB DOC) [file pone.0008938.s010.doc]

Text S1 Additional Information on the Construction of the Datasets

**Synthetic datasets**

Synthetic motif weight matrices (WMs) were constructed as in Siddharthan *et al.* [1]; for each position in the motif WM we picked a random “consensus” nucleotide, set the probability of that nucleotide to p and set the probabilities of the other nucleotides to (1-p)/3, where p is called the polarization of the motif WM. We created two different motif WMs: 1) a high IC motif WM of width 13 bp with p=0.90 for each position and 2) a more degenerated, low IC motif WM of width 13 bp with for each position p=0.75. Motif sites were sampled from both motif WMs to create input sequences containing respectively a high or low IC motif. We embedded each sampled motif site at a randomly chosen position in a background sequence of length 500 bp that was randomly generated. Each ancestral sequence (~a background sequence containing an embedded motif site) was then evolved along a phylogenetic tree under a defined evolutionary model to create phylogenetically related sequences. For the background sequence (whole sequence except the motif sites) we used the Jukes and Cantor (JC) model [2], for the embedded motif sites an adapted Felsenstein (F81) model [3]. To simulate evolution under the JC model we used the software Rose [4]. Rose creates, guided by a phylogenetic tree and an evolutionary model, a family of evolutionary related sequences starting from an ancestral sequence by insertion, deletion and substitution of characters. The branch length of the Rose input tree equals the expected number of substitutions per 100 sites (default branch length multiplied by 100). By setting the ‘mutation probability’ parameter to 1.0 for all sites in the ancestral sequence, the number of substitutions introduced for each 100 sites of the sequence on average equals the branch lengths of the tree. Of course, the number of observed mutations might be smaller due to back-mutations, especially if the branch length is large. The insertion and deletion threshold was set to zero. For the embedded motif sites we simulated the evolution according to the adapted F81 evolutionary model; we used the approach described in Siddharthan *et al.* [1]. Evolutionary related motif sites were created starting from the embedded ancestral motif site following a phylogenetic tree with proximities (q). For each position in the orthologous motif site, the probability of finding a nucleotide equal to the ancestral nucleotide is q and the probability of a mutation is (1-q). When the ancestral nucleotide was mutated, it was replaced by a new nucleotide sampled from the motif WM.

**Real datasets**

For the real data we constructed datasets containing a high IC or a more degenerated, low IC motif for both the Gamma-proteobacterial and for the *Saccharomyces* species. To construct the datasets for the Gamma-proteobacteria in the coregulation space, we selected according to RegulonDB [5], genes in *Escherichia coli* that contain at least one annotated motif site for respectively the regulators LexA and TyrR. To extend the LexA and TyrR datasets in the combined space, we searched for each of their target genes the corresponding orthologs in other Gamma-proteobacteria (*Shigella flexneri*, *Salmonella typhimurium*, *Salmonella enterica*, *Yersinia pestis*, *Erwinia carotovora*, *Vibrio Cholerae* and *Pseudomonas aeruginosa*). For the ortholog detection we used the reciprocal smallest distance approach (RSD) [6]. For the orthologous space we selected *E. coli* genes that have exactly one annotated LexA or TyrR motif site and their corresponding orthologs. For the yeast datasets in the coregulation space, we selected target genes in *Saccharomyces cerevisiae* for respectively the regulators URS1H and RAP1 based on annotated motif sites in SCPD [7] and SwissRegulon [8]. Corresponding orthologs for all target genes were retrieved from the *Saccharomyces* Genome Database (SGD project. "*Saccharomyces* Genome Database" <http://www.yeastgenome.org/>, accessed 14 March 2009) in case of *Saccharomyces paradoxus*, while for *Saccharomyces mikatae*, *Saccharomyces kudriavzevii* and *Saccharomyces bayanus* we used data from the Washington University group [9]. For the orthologous space we selected *S. cerevisiae* genes that have exactly one annotated URS1H or RAP1 motif site and their corresponding orthologs. Table S2 gives the exact composition for each real dataset.

Reference List

1. Siddharthan R, Siggia ED, van Nimwegen E (2005) PhyloGibbs: a Gibbs sampling motif finder that incorporates phylogeny. PLoS Comput Biol 1: e67.

2. Jukes TH, Cantor CR (1969) Evolution of protein molecules. In: Mammalian protein metabolism. New York: Academic Press. pp. 21-132.

3. Sinha S, van Nimwegen E, Siggia ED (2003) A probabilistic method to detect regulatory modules. Bioinformatics 19 Suppl 1:I292-I301.: I292-I301.

4. Stoye J, Evers D, Meyer F (1998) Rose: generating sequence families. Bioinformatics 14: 157-163.

5. Huerta AM, Salgado H, Thieffry D, Collado-Vides J (1998) RegulonDB: a database on transcriptional regulation in *Escherichia coli*. Nucleic Acids Res 26: 55-59.

6. Wall DP, Fraser HB, Hirsh AE (2003) Detecting putative orthologs. Bioinformatics 19: 1710-1711.

7. Zhu J, Zhang MQ (1999) SCPD: a promoter database of the yeast Saccharomyces cerevisiae. Bioinformatics 15: 607-611.

8. Pachkov M, Erb I, Molina N, van NE (2007) SwissRegulon: a database of genome-wide annotations of regulatory sites. Nucleic Acids Res 35: D127-D131.

9. Cliften P, Sudarsanam P, Desikan A, Fulton L, Fulton B, et al. (2003) Finding functional features in *Saccharomyces* genomes by phylogenetic footprinting. Science 301: 71-76.

10. Guindon S, Gascuel O (2003) A simple, fast, and accurate algorithm to estimate large phylogenies by maximum likelihood. Syst Biol 52: 696-704.

11. Newberg LA, Thompson WA, Conlan S, Smith TM, McCue LA, et al. (2007) A phylogenetic Gibbs sampler that yields centroid solutions for cis-regulatory site prediction. Bioinformatics 23: 1718-1727.
