# Supplementary material for: The Effect of Orthology and Coregulation on Detecting Regulatory Motifs
Source: PLoS One. 2010 Feb 3;5(2):e8938. doi: 10.1371/journal.pone.0008938 (PMC2815771; doi:10.1371/journal.pone.0008938)
Supplement: Text S2 — provides additional information on the pre-processing of the alignments. (0.07 MB DOC) [file pone.0008938.s011.doc]

Text S2 Pre-processing of the Alignments

Both phylogenetic algorithms need as input a prealignment of the orthologous sequences in order to account for their phylogenetic relatedness by the use of an evolutionary model. In this section of the supplementary we explain why we use different alignment strategies for PG and PS and we describe the results for using PS in combination with the local alignment strategy Dialign.

**The different prealignment strategies used by PG and PS**

As also mentioned in Table S1, PG and PS use a different alignment strategy: PS makes use of a global alignment strategy (ClustalW [1]) while PG uses a local one (Dialign [2]). Being a global alignment strategy, ClustalW enforces the alignment to span the entire length of the sequences and by definition aligns all sequences. A ClustalW alignment thus contains the conserved regions in well aligned blocks, while the unconserved parts are usually located in ill-aligned, gapped regions. Because of these intrinsic properties, such global alignment strategies underperform when further related sequences and/or sequences with unequal length are included [3]. The local alignment strategy Dialign identifies and aligns local regions of similarity within the sequences (aligned regions) and leaves the less conserved regions or sequences unaligned. Dialign thus explicitly annotates aligned and unaligned regions differently. Aligned regions can also cover a subset of the sequences only. It usually outperforms global strategies when unconserved sequences of unequal length are included.

The reason why both algorithms rely on different strategies to generate these prealignments stems from the difference in the way they use these prealignments. PS can only cope with global prealignments: from these global prealignments only regions that are gaplessly aligned over all species in the prealignment are considered as potential motif sites (also called **blocks**). Unaligned regions (corresponding to the gapped regions in a global prealignment) are ignored in the further analysis. For PG on the other hand, both the gaplessly aligned regions and unaligned regions are considered as potential motif sites, also called windows. For PG a window thus can contain a set of orthologous sites (**multi-species window**) as well as a single unaligned, independent site (**single-species window**). PG treats each of these subparts of the prealignment differently: for multi-species windows the phylogenetic relatedness between the sequences is taken into account while the single-species windows are treated independently. PG thus benefits from using annotated, local prealignments as input. Figure A clarifies the terms ‘windows’ and ‘blocks’.


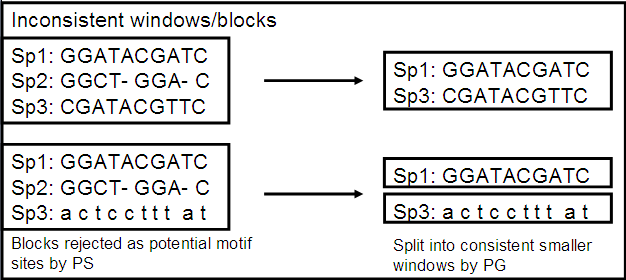


**Figure A** Explains the terms windows (used by PG) and blocks (used by PS).

Both PG and PS have to assign sets of putative orthologous motif sites to a prealignment of orthologous sequences and these sets of putative orthologous motif sites are called windows (PG) or blocks (PS). When such a set of putative orthologous motif sites is not perfectly aligned over all sequences (contains gaps or unaligned parts) it is called an inconsistent window (PG) or an inconsistent block (PS). On the left of Figure A, two examples of such an inconsistent window/block are shown: putative orthologous motif sites aligned over three sequences from respectively three species (Sp1, 2 and 3) that contain gaps and unaligned parts. PS does not consider inconsistent blocks for further analysis as they are rejected as potential motif sites. PG in contrast can split up inconsistent multi-species windows into smaller consistent windows that contain no gaps/unaligned parts by leaving out the ill aligned/unaligned sequences (this is done automatically by setting parameter –D to one (see section 4: ‘Parametersettings’)). These smaller consistent windows can still be multi-species windows (first example) that will be scored by an evolutionary model or they can become single-species windows (second example) that will be treated as independent motif sites.

**Tests in which PS was used in combination with the local alignment strategy Dialign**

It is in principle also possible to use Dialign instead of ClustalW in combination with PS. For datasets that contain easy to align sequences (e.g. the synthetic datasets with sequences of equal length) the global and local alignment strategies perform equally well and result in similar high quality alignments. So in those cases there is no difference between using ClustalW or Dialign. For the more difficult to align sequences (which in our analysis corresponded to the bacterial and yeast datasets that consisted of intergenic sequences of different length), the local alignment strategy can perform better than a global one.

To rule out that for those difficult to align sequences, the difference in performance we observed between PG and PS was due to the difference in the used alignment procedure rather than in the intrinsically different way both algorithms handle the alignments, we also run tests of PS with alignments obtained by Dialign (results in Table A below). When doing so, the regions that are well aligned over all sequences are extracted from the local prealignment prior to providing them as input to PS. Using a local alignment strategy did not improve the results obtained by PS compared to using ClustalW. When using a local alignment strategy on those difficult to align datasets most regions will be left unaligned which usually improves the quality of the prealignment. However, as PS can only search for motifs in the regions that are well aligned over all orthologs, the prealignment does not longer contain input information for PS. Therefore, it is often more advantageous to use PS in combination with ClustalW than with Dialign (which we therefore did in the remainder of the analysis).

**Table A** Results of PS when using a local alignment strategy on the difficult to align real datasets in the combined coregulation-orthology space.

| GAMMA-PROTEOBACTERIA | | | | | | | | |
| --- | --- | --- | --- | --- | --- | --- | --- | --- |
| SETUP | HIGH IC - LexA | | | | LOW IC - TyrR | | | |
| **Results of PS** | | | | | | | | |
| **# orthologs = 6** | **R1** | **RR** | **spPPV** | **spSens** | **R1** | **RR** | **spPPV** | **spSens** |
| *Global alignment strategy* | *10* | *90* | *69* | *42.4* | *10* | *100* | *85* | *36.4* |
| Local alignment strategy | 10 | 100 | 100 | 33.6 | 0 | / | / | / |
| *SACCHAROMYCES* SPECIES | | | | | | | | |
| SETUP | HIGH IC – URS1H | | | | LOW IC – RAP1 | | | |
| **Results of PS** | | | | | | | | |
| **# orthologs = 5** | **R1** | **RR** | **spPPV** | **spSens** | **R1** | **RR** | **spPPV** | **spSens** |
| *Global alignment strategy* | *10* | *100* | *84* | *75.5* | *10* | *100* | *88.8* | *79* |
| Local alignment strategy | 10 | 100 | 16 | 11.8 | 0 | / | / | / |

**Performance and quality measures: R1**: the number of runs with an output out of the 10 runs on one real dataset, **RR (%)**: Recovery Rate: the percentage of the output (R1) for which the correct motif was retrieved (correct outputs), **spPPV (%)**: species-dependent PPV: the percentage of true sites among the predicted sites for the reference species, averaged over all correct outputs, **spSens (%)**: species-dependent Sens: the percentage of the true sites in the reference species found by the algorithm, averaged over all correct outputs. The reference species equals *E. coli* or *S. cerevisiae*. **Gamma-proteobacteria:** The dataset of each regulator consists of 8 (LexA) or 7 (TyrR) target genes from the reference species (Table S2), together with their orthologs in 5 additional species (Figure S1 lists from which species these orthologs were derived). Each reference sequence together with its orthologs was prealigned so in total we have 6 prealigned orthologs, related trough the neutral tree for the Gamma-proteobacteria. ***Saccharomyces* species:** The dataset of each regulator (URS1H and RAP1) consists of 10 target genes from the reference species (Table S2), together with their orthologs in 4 additional species (Figure S1 lists from which species these orthologs were derived). Each reference sequence together with its orthologs was prealigned so in total we have 5 prealigned orthologs, related trough the neutral tree for the *Saccharomyces* species. Table S4 shows the Newick formats of both trees. We provide **as input to PS** the regions of a local prealignment (Dialign, threshold = 2) that were conserved over all 6 or 5 orthologs. As *reference* also the results of PS are given that were obtained with the full ClustalW prealignment.

Reference List

1. Chenna R, Sugawara H, Koike T, Lopez R, Gibson TJ, et al. (2003) Multiple sequence alignment with the Clustal series of programs. Nucleic Acids Res 31: 3497-3500.

2. Morgenstern B (1999) DIALIGN 2: improvement of the segment-to-segment approach to multiple sequence alignment. Bioinformatics 15: 211-218.

3. Van Hellemont R, Monsieurs P, Thijs G, De Moor B, Van de PY, et al. (2005) A novel approach to identifying regulatory motifs in distantly related genomes. Genome Biol 6: R113.
