# Supplementary material for: The Effect of Orthology and Coregulation on Detecting Regulatory Motifs
Source: PLoS One. 2010 Feb 3;5(2):e8938. doi: 10.1371/journal.pone.0008938 (PMC2815771; doi:10.1371/journal.pone.0008938)
Supplement: Text S3 — provides additional information on the parametersettings. (0.08 MB DOC) [file pone.0008938.s012.doc]

Text S3 Additional Information on the Parametersettings

The parametersettings used for MEME were mentioned in the “Material and Methods” section of the main text. As both phylogenetic algorithms have some more specific parameters to integrate e.g. the phylogeny, we discuss their parameters in more detail in this section. Table A shows the parametersettings used in this study for PG and PS for all performed tests on synthetic and real datasets and is followed by a description of these parameters.

**Table A** The parametersettings for PG and PS on the synthetic and real datasets.

| **PG** | | | |
| --- | --- | --- | --- |
| **Parameter** | **Symbol** | **Synthetic datasets** | **Real datasets** |
| Input tree | -L | Phylogenetic tree in Newick format, distances given by proximities | Gamma-proteobacterial tree or *Saccharomyces* species tree both with proximities |
| Order of Markov model for background probabilities | -N | -1 | 1 (default) |
| Alignment level | -D | 0: unaligned sequences  1: aligned sequences | 0: unaligned sequences  1: aligned sequences |
| Motif width | -m | 13 | 20 (LexA)  18 (TyrR)  13 (URS1H)  10 (RAP1) |
| Expected number of windows (unless mentioned differently in tables) | -I | - unaligned: number of embedded motif sites in the input data  - aligned/partially aligned: number of embedded motif sites in the input data divided by the number of orthologs per gene | -unaligned: number of annotated motif sites present in *E. coli*/*S. cerevisiae* multiplied by the number of orthologs per gene  -aligned: number of annotated motif sites present in *E.coli/S. cerevisiae* |
| Palindromic motif | -C | No palindromic motif sites | -No C for LexA, URS1H and RAP1  -C for TyrR (see Table B) |
| Tracking threshold  (unless mentioned differently in tables) | -E | 0.50 | 0.50 |
| Number of cycles during annealing/tracking | -S | 100 | 100 |
| Reverse complement | -r | Use r (only search on the forward strand) | Use r (only search on the forward strand) |
| **PS** | | | |
| **Parameter** | **Symbol** | **Synthetic datasets** | **Real datasets** |
| Input tree | Tree* | For each MASS a separate phylogenetic tree relating only the species used in that MASS. Newick format with distances given by branch lengths. | For each MASS a Gamma-proteobacterial tree or a *Saccharomyces* species tree, only relating the species for that MASS (branch lengths). |
| Sequences weights | Weights* | Made by Seq.weights.pl | Made by Seq.weights.pl |
| Background composition model | -B | Made by unifiedcpp.opteron | Made by unifiedcpp.opteron |
| Alignment of the centroid motif sites | -Align_centroid | Use Align_centroid | Use Align_centroid |
| Reverse complement | -r | Use r (only search on the forward strand) | Use r (only search on the forward strand) |
| Palindromic motif | -R | No palindromic motif sites | No –R for LexA, URS1H and RAP1  -R 1,1,9 for TyrR (see Table B) |
| Motif width | / | 13 | 20 (LexA)  18 (TyrR)  13 (URS1H)  10 (RAP1) |
| Expected number of motif sites (unless mentioned differently in tables) | / | Number of embedded motif sites in the input data | Number of annotated motif sites in *E. coli/S. cerevisiae* multiplied by the number of orthologs per gene |
| Maximum number of motif sites per sequence | -E | 1 | Maximum number of annotated motif sites present in one of the genes in the input file |
| Prior distribution on the number of motif sites per sequence | Blocks* | 0.50 0.95 | No prior info |
| Bayesian sampling | -bayes | 2000,8000 | 2000,8000 |
| Number of seeds (re-initializations) | -S | 20 | 20 |
| Nucleotide alphabet | -n | Use n | Use n |

*For PS the prior information is gathered in a ‘prior file’ (-P). In this prior file, terms as ‘Tree’, ‘Weights’ and ‘Blocks’ are used to specify the parameter for which additional information was provided (see also in the description of the parameters).

**The description of the parameters of both phylogenetic motif detection algorithms**

**Parameters describing the motifs**: both algorithms need an initial guess on *the expected number of motifs* present in the input sequences and for each motif *the expected number of motif sites*. This information is captured in the parameter –I for PG and for PS this information is mentioned just after the program name in the command line. The format to describe this information is for both algorithms the same, e.g. ‘10,10’ when we expect two different motifs, each with 10 expected motif sites. In this work in principle all described datasets contain exactly one motif. Both algorithms were therefore asked to search exactly for one motif model per dataset. For PG, the expected number of motif sites is described by the expected number of ‘windows’ (see Table S1: ‘Motif model’). For a dataset that contains only unaligned sequences, a window always equals a single motif site (~single-species windows) while for a dataset with prealigned sequences, a window equals or a single unaligned motif site or a set of multiple aligned motif sites (~multi-species windows). So to define the expected number of windows the user has to take into account if the sequences in the dataset are prealigned or not. For unaligned sequences the expected number of windows equals the total number of embedded motif sites for the synthetic data and the number of motif sites in *E.coli/S. cerevisiae* multiplied by the number of orthologs for the real data (assuming that all orthologous genes contain the same number of motif sites). For prealigned sequences the expected number of windows was set to the number of embedded motif sites divided by the number of orthologs for the synthetic data and for the real data to the number of motif sites in *E. coli/S. cerevisiae*.

For PS, the expected number of motif sites present in the input sequences was chosen equal to the total number of embedded motif sites for the synthetic data and for the real data equal to the number of motif sites in *E. coli/S. cerevisiae* multiplied by the number of orthologs. PS has two extra parameters compared to PG concerning prior information on the number of motif sites per sequence. The first parameter is -E, *the maximum number of motif sites per sequence*. This parameter was set to one for the synthetic data and equal to the highest number of motif sites present in one of the input sequences for the real data. The second parameter describes *the prior probabilities for finding zero, one until -E sites per sequence*. We set the prior probabilities for finding zero and one motif site per sequence to respectively 0.50 and 0.95 for the synthetic data, while for the real data we used uniform probabilities to find zero, one until -E motif sites per sequence. For the synthetic data a prior probability for finding zero motif sites per sequence equal to 0.50 had a positive influence in the presence of noisy sequences (sequences without motif sites), but did not deteriorate the performance of detecting the true motifs in the absence of noise (data not shown). This prior information is provided to the algorithm through a prior file (–P) that can contain different types of prior information each labeled by a short term, in this case ‘>Blocks’.

Other prior information on the motif is the *motif width* (number of conserved motif positions): for both algorithms this parameter equaled the total motif length (13 bp for the synthetic data, 20 bp respectively 18 bp for LexA and TyrR and 13 bp respectively 10 bp for URS1H and RAP1). This motif width is not restrictive for PS, because for the default settings of the algorithm the *fragmentation option* -F is turned on which means that PS allows conserved motif positions to be interrupted by degenerated motif positions and as such alters and optimizes the length of the motif.

All algorithms allow the option to search for special motif types such as palindromic motifs. In Table B (beneath) we tested the effect of using a specific *model for palindromic motifs* or not, on the real LexA and TyrR datasets. We found that for both phylogenetic algorithms, the TyrR datasets gave the best results when choosing for a palindromic model, while for the LexA datasets the opposite was true (probably because the degenerate spacer has less palindromic properties). So for the TyrR datasets we used parameter –C for PG and parameter -R1,1,9 for PS (-R1,1,9 indicates that the first (1) motif model we search for is palindromic in positions 1 through 9, implying automatically a corresponding position, the same distance away from the opposite end of the motif that is also palindromic). For the synthetic and yeast data (URS1H and RAP1), the motif sites were all non-palindromic.

To search for motifs on one strand only and not on the complementary strand we used parameter –r for both algorithms.

**Table B** The effect of using a palindromic motif model for PG, PS and MEME when searching for the LexA and TyrR motif in the **Gamma-proteobacterial** datasets in the coregulation space.

| GAMMA-PROTEOBACTERIA | | | | | | | | |
| --- | --- | --- | --- | --- | --- | --- | --- | --- |
| SETUP | HIGH IC - LexA | | | | LOW IC - TyrR | | | |
| **Results of PG** | | | | | | | | |
| **Model** | **R1** | **RR** | **spPPV** | **spSens** | **R1** | **RR** | **spPPV** | **spSens** |
| Palindromic | 9 | 100 | 96.4 | 79.8 | 8 | 100 | 92 | 58.3 |
| Non Palindromic | 10 | 100 | 98 | 81.8 | 5 | 80 | 90.7 | 58.3 |
| **Results of PS** | | | | | | | | |
| **Model** | **R1** | **RR** | **spPPV** | **spSens** | **R1** | **RR** | **spPPV** | **spSens** |
| Palindromic | 10 | 100 | 88.9 | 72.7 | 10 | 100 | 100 | 57.1 |
| Non Palindromic | 10 | 100 | 100 | 81.8 | 10 | 100 | 100 | 44 |
| **Results of MEME** | | | | | | | | |
| **Model** | **R1** | **RR** | **spPPV** | **spSens** | **R1** | **RR** | **spPPV** | **spSens** |
| Palindromic | 10 | 100 | 90.9 | 90.9 | 10 | 100 | 73.3 | 73.3 |
| Non Palindromic | 10 | 100 | 81.8 | 81.8 | 10 | 100 | 73.3 | 73.3 |

**Performance and quality measures: R1**: the number of runs with an output out of the 10 runs on one real dataset, **RR (%)**: Recovery Rate: the percentage of the output (R1) for which the correct motif was retrieved (correct outputs), **spPPV (%)**: species-dependent PPV: the percentage of true sites among the predicted sites for the reference species, averaged over all correct outputs, **spSens (%)**: species-dependent Sens: the percentage of the true sites in the reference species found by the algorithm, averaged over all correct outputs. *E. coli* is the reference species. **Model:** ‘Palindromic’ stands for using the palindromic parameters (PG: -C, PS: –R 1,1,10 for LexA and –R 1,1,9 for TyrR and MEME: -pal), ‘Non Palindromic’ stands for no use of the palindromic parameters. **Gamma-proteobacteria:** The dataset of each regulator consists of 8 (LexA) or 7 (TyrR) target genes in *E. coli* (Table S2).

**Parameters influencing the search algorithm:** The PG algorithm can be split up in simulated annealing and tracking (Table S1: ‘Sampling’), *the number of cycles* for each phase (–S) was set to the default value of 100. One of the parameters often mentioned in the results section is the *tracking threshold* –T, which determines the trade-off between sensitivity and PPV (it corresponds to the frequency with which motif sites are co-sampled with the reference configuration during the tracking cycles). By increasing the default threshold of 0.05 to 0.50 we observed a drastic increase in PVV at the expense of only a slight drop in sensitivity, resulting in a better overall performance (data not shown). Unless indicated explicitly we always used T= 0.50. PS consists of burn-in and *sampling iterations* (Table S1: ‘Sampling’) respectively set to 2000 and 8000 iterations (-bayes). The number of re-initializations (-S) was set to 20, to avoid that the algorithm reports a local optimum as is the case for low values of –S. When using *the fragmentation option* –F (see paragraph motif width) we also turned on the option *-align_centroid* to obtain a motif WM (this to align the centroid motif sites of different length resulting from the –F option). For PS the stringency of the centroid sites can not be altered by the user.

**Parameters relating to the use of the phylogeny** **when working with prealigned orthologous sequences.** Both algorithms need as input a *phylogenetic tree* in Newick format with distances described by proximities for PG and branch lengths for PS. PS requires a separate tree for each MASS containing only the species present in this particular set of aligned orthologs (provided through the prior file –P indicated by ‘>TREE’). The provided perl script (Seq.weights.pl) was used to calculate the sequence weights (Table S1: ‘Scoring’) for each MASS based on the corresponding tree. The obtained sequence weights were provided to the algorithm through the prior file, indicated by ‘>WEIGHTS’. PG works with one tree relating all species present in the input dataset. An additional parameter in the PG algorithm is *the alignment level* (-D). This parameter indicates if the sequences in the dataset are prealigned (-D=1 or -D=2) or not (-D=0). For prealigned sequences the user can specify by using –D the placement of the windows: -D=1 (splits up multi-species windows containing gaps into smaller windows without gaps) or –D=2 (gapped windows will be left out) (see also Text S2). For both the synthetic and real data –D was set to zero for the unaligned data and to one for the prealigned data.

**Parameters describing the background model:** PG uses an *N^th^ order Markov model* (–N). For the synthetic data we used (-N=-1) indicating a single nucleotide frequency of 0.25 for A, C, G and T. For the real data we tested different background models: Markov models trained on the input sequences with order 0, 1 and 3 and a single nucleotide background model derived from the full *E. coli/S. cerevisiae* genome. Except for the Markov model order 3 (for which the input sequences were not sufficiently long to calculate reliably the correlated counts), all other background models derived from the input sequences gave comparable results (data not shown). For further tests on the real data, we used the default order 1 background model derived from the input sequences. PS uses a special *position specific background model* that gives the probabilities of observing each of the four nucleotides at each position in the sequence. This background model is derived by running a Bayesian segmentation algorithm (unifiedcpp) provided by PS, on the set of input sequences (for both the real and the synthetic datasets). The generated background model was provided to the algorithm by parameter –B.
